# Validation of NBD-coupled taurocholic acid for intravital analysis of bile acid transport in liver and kidney of mice

**DOI:** 10.17179/excli2024-7707

**Published:** 2024-10-30

**Authors:** Ahmed Ghallab, Sebastian Kunz, Celine Drossel, Veronica Billo, Adrian Friebel, Mats Georg, Richard Göttlich, Zaynab Hobloss, Reham Hassan, Maiju Myllys, Abdel-latief Seddek, Noha Abdelmageed, Paul A. Dawson, Erik Lindström, Stefan Hoehme, Jan G. Hengstler, Joachim Geyer

**Affiliations:** 1 Department of Toxicology, Leibniz Research Centre for Working Environment and Human Factors, Technical University Dortmund, Ardeystr. 67, 44139 Dortmund, Germany; 2 Department of Forensic Medicine and Toxicology, Faculty of Veterinary Medicine, South Valley University, 83523 Qena, Egypt; 3 Institute of Pharmacology and Toxicology, Justus Liebig University Giessen, Biomedical Research Center Seltersberg, Schubertstr. 81, 35392 Giessen, Germany; 4 Institute of Organic Chemistry, Justus Liebig University Giessen, Heinrich-Buff-Ring 17, 35392, Giessen, Germany; 5 Institute of Computer Science & Saxonian Incubator for Clinical Research (SIKT), University of Leipzig, Haertelstraße 16-18, 04107 Leipzig, Germany; 6 Department of Pharmacology, Faculty of Veterinary Medicine, Sohag University, 82524 Sohag, Egypt; 7 Department of Pediatrics, Division of Gastroenterology, Hepatology, and Nutrition, Emory University, Atlanta, GA 30322, USA; 8 Albireo Pharma, Inc., Boston, MA 02109, USA

**Keywords:** Ntcp, OATP, ASBT, cholestasis, cholemic nephropathy

## Abstract

Fluorophore-coupled bile acids (BA) represent an important tool for intravital analysis of BA flux in animal models of cholestatic diseases. However, addition of a fluorophore to a BA may alter transport properties. We developed and validated a 4-chloro-7-nitrobenzo-2-oxa-1,3-diazole-coupled taurocholic acid (3β-NBD-TCA) as a probe for intravital analysis of BA homeostasis. We compared transport of 3β-NBD-TCA to [3H]-TCA in HEK293 cells stably expressing the mouse hepatic or renal BA carriers mNtcp or mAsbt, respectively. We also studied distribution kinetics intravitally in livers and kidneys of anesthetized wildtype and mOatp1a/1b cluster knockout mice (OatpKO) with and without administration of the Ntcp inhibitor Myrcludex B and the ASBT inhibitor AS0369. In vitro, 3β-NBD-TCA and [3H]-TCA showed comparable concentration- and time-dependent transport via mNtcp and mAsbt as well as similar inhibition kinetics for Myrcludex B and AS0369. Intravital analysis in the livers of wildtype and OatpKO mice revealed contribution of both mNtcp and mOatp1a/1b in the 3β-NBD-TCA uptake from the sinusoidal blood into hepatocytes. Combined deletion of mOatp1a/1b and inhibition of mNtcp by Myrcludex B blocked the uptake of 3β-NBD-TCA from sinusoidal blood into hepatocytes. This led to an increase of 3β-NBD-TCA signal in the systemic circulation including renal capillaries, followed by strong enrichment in a subpopulation of proximal renal tubular epithelial cells (TEC). The enrichment of 3β-NBD-TCA in TEC was strongly reduced by the systemic ASBT inhibitor AS0369. NBD-coupled TCA has similar transport kinetics as [3H]-TCA and can be used as a tool to study hepatorenal BA transport.

See also the graphical abstract(Fig. 1).

## Introduction

Recently, intravital 2-photon imaging opened new possibilities to study the uptake and secretion process of endogenous metabolites (Ghallab et al., 2021[23][25], 2022[22]; Hassan, 2016[29]; Reif et al., 2017[54]; Remetic et al., 2022[55]) and xenobiotics (Ghallab et al., 2021[25]; Hassan et al., 2022[30], 2024[31]). This technique allows the time-resolved analysis of tissue compartments, such as the sinusoidal (blood), hepatocellular and bile canalicular compartments of the liver (Ghallab et al., 2022[22]) as well as renal capillaries, tubular lumen, and tubular epithelial cells of the kidney (Ghallab et al., 2024[21]; Koeppert et al., 2021[33]). Intravital 2-photon imaging depends on the detection of the fluorescence signal of the studied compound. Only a few metabolites and xenobiotics show sufficient autofluorescence for this technique (Ghallab et al., 2021[23]). For most substances, intravital imaging depends on the coupling of the analyzed metabolites to fluorophores (Marques et al., 2015[44]; Wang et al., 2024[68]). However, addition of a fluorophore affects the structure and physicochemical properties of the metabolite and thereby may influence transport properties by cellular carriers. 

Intravital imaging of bile acids (BA) allowed the direct visualization of pathomechanisms of cholestatic diseases, such as the paracellular leakage of BA from canaliculi into sinusoidal blood (Ghallab et al., 2022[22]), apical hepatocyte membrane rupture events in bile infarcts (Ghallab et al., 2019[24]) or BA enrichment in renal tubular epithelial cells (TEC) (Ghallab et al., 2024[21]). Several fluorophore-coupled BA, where the fluorescent substance is coupled to the BA side chain, have been previously introduced to study the enterohepatic transport of BA (Holzinger et al., 1997[32]; Maglova et al., 1995[41]; Milkiewicz et al., 2001[45]; Rohacova et al., 2009[56]; Schramm et al., 1991[59]; Stieger, 2022[62]). Also, for the analyzed 3β-NBD-TCA, efficient hepatobiliary excretion in a rat in situ liver perfusion model has previously been shown, comparable to the parent TCA (Petzinger et al., 1999[52]; Schramm et al., 1991[59]). However, to our knowledge detailed analysis of the respective transporters for these BA acid derivatives to validate their use for intravital imaging is lacking. A validation of fluorophore-coupled BA for intravital imaging requires an *in vitro* quantitative comparison of the transport characteristics for the transporters of interest by using the native and fluorophore-coupled BA molecules. Moreover, a validation *in vivo* is required to guarantee that the fluorophore-coupled BA shows similar systemic kinetics and transporter dependencies as the native BA. Leuenberger et al. (2021[38]) characterized the transport properties of cholic and chenodeoxycholic acid after conjugating small organic dyes to the side chain and showed that they were transported by OATPs, but they are not substrates of Ntcp or ASBT, the main BA uptake transporters in the liver and gut, respectively. Moreover, the widely used fluorescent BA analogue, cholyl‐L-lysyl‐fluorescein (CLF), is also a substrate for OATPs and MRP2 but not for Ntcp or ASBT(de Waart et al., 2010[12]). The goal of the present study was to investigate the transport of a 4-chloro-7-nitrobenzo-2-oxa-1,3-diazole-coupled taurocholic acid (3β-NBD-TCA) *in vitro* and *in vivo* to validate it as a probe for intravital analysis of BA homeostasis in the entero-nephro-hepatic circulation. We focused on the hepatic mouse BA transporters mNtcp (Na+/taurocholate co-transporting polypeptide, gene symbol Slc10a1) and mOatp1a/1b (organic anion transporting polypeptides from the Oatp1a and 1b subfamilies, gene cluster symbol Slco1a/1b) that together are responsible for the uptake of conjugated BA from sinusoidal blood into hepatocytes (Slijepcevic et al., 2017[61]). In addition, we analyzed the role of the mouse transporter mAsbt (apical sodium-dependent bile acid transporter, gene symbol Slc10a2) that besides its role for BA uptake into cholangiocytes in the liver and ileal enterocytes in the intestine, is also responsible for uptake of BA from the renal tubule lumen into TEC (Christie et al., 1996[9]; Craddock et al., 1998[10]; Lack, 1979[37]). 

## Materials and Methods

### Chemicals

All chemicals, unless otherwise stated, were purchased from Sigma-Aldrich (St. Louis, MO, USA). [^3^H]-labeled taurocholic acid ([^3^H]-TCA, 20 Ci/mmol, 0.09 mCi/mL) was purchased from PerkinElmer Life Sciences (Waltham, MA, USA).

### Cloning of mouse Abst (mAsbt) and mouse Ntcp (mNtcp)

RNA was isolated from mouse ileum and liver and reverse transcribed to cDNA with SuperScript III Reverse Transcriptase (Invitrogen) as reported before (Geyer et al., 2007[20]). The organ samples represent surplus material from a former animal study that has been approved by the local regulatory authority (Regierungspraesidium Giessen) with the reference number V54-19 c 20 15 h 02 GI 20/23 Nr. A8/2013 (Bakhaus et al., 2018[2]). The full open reading frames for mAsbt and mNtcp were amplified using Phusion Flash PCR Master Mix (F-548, Thermo Scientific) with the following primers: 5'-ATA AAG CTT GCC ACC ATG GAT AAC TCC TCT GTC TG-3' forward and 5'-ATA CTC GAG CTA CTT ATC GTC GTC ATC CTT GTA ATC CTT CTC ATC TGG TTG AAA TCC CTT GTT TGT-3' reverse for mAsbt, and 5'-ATA AAG CTT GCC ACC ATG GAG GCG CAC AAC GTA TC-3' forward and 5'-ATA CTC GAG CTA CTT ATC GTC GTC ATC CTT GTA ATC ATT TGC CAT CTG ACC AGA GTT CAG GCC ATT-3' reverse for mNtcp. Via the reverse primers the FLAG-epitope (FLAG sequence underlined) was introduced in-frame at the 3' end of the carrier constructs. PCR amplification was performed on a peqSTAR XS PEQLAB PCR cycler for 30 cycles under the following conditions: initialization for 5 min at 95 °C, denaturation for 30 s at 95 °C, annealing for 30 s at 60 °C, extension for 90 s at 72 °C, final elongation for 5 min at 72 °C and final hold at 8 °C. After amplification the PCR products of 1029 bp (mAsbt) and 1102 bp (mNtcp) were separated by 1 % agarose gel electrophoresis (Supplementary Figure 1) and the appropriate bands were excised and purified with the GeneJET PCR Purification Kit (#K0702, Thermo Scientific). The PCR products were then cloned into the pcDNA5 expression vector (Invitrogen) via restriction sites *Hin*dIII and *Xho*I. Final constructs (mNtcp-FLAG and mAsbt-FLAG) were sequence verified by DNA sequencing (Seqlab Microsynth).

### Generation of mNtcp-HEK293 and mAsbt-HEK293 stable cell lines

T-Rex Flp-In HEK293T cells (Thermo Fisher Scientific, Waltham, MA, USA) were used to generate stably transfected tetracycline-inducible HEK293 cell lines that express the mNtcp-FLAG and mAsbt-FLAG proteins, respectively, after treatment with tetracycline. Briefly, the pcDNA5 mNtcp-FLAG and mAsbt-FLAG constructs were co-transfected with the Flp recombinase coding plasmid pOG44 (Invitrogen, Carlsbad, CA, USA). Stable integration of the expression plasmid was achieved through homologous recombination at the Flp recombinase target (FRT) site in the plasmid and cell genome. Stable cell clones were selected for hygromycin (150 µg/mL) resistance for several weeks. The parental cell line and all stable cell clones were maintained at 37 °C, 5 % CO_2_, and 95 % humidity in DMEM/F-12 medium (PAA, Pasching, Germany) supplemented with 10 % fetal calf serum, 4 mM L-glutamine (PAA, Pasching, Germany) and penicillin/streptomycin (PAA, Pasching, Germany). Proper integration of the mNtcp-FLAG and mAsbt-FLAG constructs into the HEK293 cell genome was verified by Sanger sequencing (Microsynth AG, Balgach, Switzerland) and mRNA expression of the transgene was verified by PCR amplification (Supplementary Figure 1) and immunofluorescence staining. The resulting stable cell lines are further referred to as mNtcp-HEK and mAsbt-HEK, respectively. 

### Immunofluorescence detection of mAsbt-FLAG and mNtcp-FLAG expression in HEK293 cells

The mNtcp-HEK and mAsbt-HEK cells were seeded (1.0 x 10^4^ per well) into 8-well µ-slides (80826; IBIDI, Graefelfing, Germany) coated with poly-L-lysine. Expression of the recombinant proteins was induced by tetracycline treatment (1 µg/mL) for 72 h. Then, cells were fixed for 15 min with 3 % paraformaldehyde (PFA) at room temperature. Thereafter, cells were washed with PBS (137 mM NaCl, 2.7 mM KCl, 1.5 mM KH_2_PO_4_, 7.3 mM Na_2_HPO_4_, pH 7.4, 37 °C) and consequently permeabilized in the presence of 0.2 % triton X for 30 min. After washing, cells were blocked with 5 % bovine serum albumin in PBS for 30 min at room temperature. For detection of the FLAG-tag, mNtcp-HEK and mAsbt-HEK cells were incubated with an anti-FLAG antibody (1:300 dilution; produced in rabbit; Sigma #F7425; 0.8 mg/ml stock) for 1 h at 37 °C. After washing three times, cells were incubated with the secondary anti-rabbit antibody (1:800 dilution; produced in goat; IgG-Alexa Flour488; Life Technologies; 2mg/ml stock) and the nucleus dye Hoechst (1:1000) for 1 h at 37 °C protected from light. After washing three times with PBS, immunofluorescence studies were performed at room temperature on an inverted Leica DM5500 fluorescence microscope. Images were generated using at 63x and 40x resolution with green (488 nm) and blue (352 nm) filter sets, respectively.

### Synthesis of 3β-NBD-TCA

**Figure 2:**
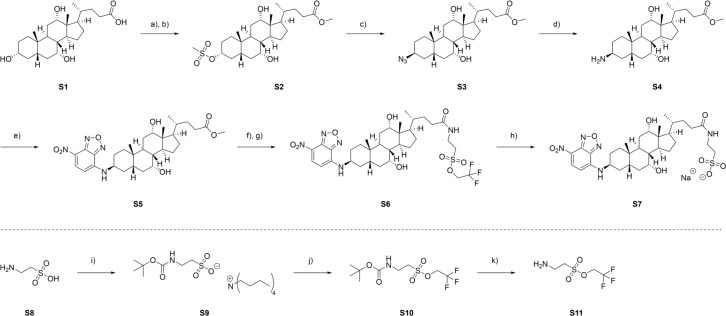
Synthesis of 3β-NBD-TCA: a) SOCl_2_, dry MeOH, 0 °C → rt, 18 h; b) Et_3_N, CH_3_SO_2_Cl, dry DCM, 0 °C, 2 h; c) NaN_3_, dry DMF, 80 °C, 48 h; d) PPh_3_, H_2_O, THF, 50 °C, 18 h; e) NBDCl, NaHCO_3_, MeOH, 50 °C, 18 h; f) 2N LiOH, MeOH, 40 °C, 3 h; g) Et_3_N, TBTU, HOBt, dry DMF, 45 min, then S11 in DMF, rt, 18 h; h) 2N NaOH in MeOH, DCM, rt, 3 h; i) 40 % aq. *n*Bu_4_NOH in H_2_O, Boc_2_O, acetone, rt, 18 h; j) (COCl)_2_ in dry DCM, dry DMF, 0 °C, 1 h, then Et_3_N, CF_3_CH_2_OH in dry DCM, rt, 18 h; k) TFA, DCM, rt, 4 h.

The 3β-NBD-TCA (7α,12α-dihydroxy-3β-[(7-nitro-2,1,3-benzoxadiazol-4-yl)amino]-5β-oxocholan-24yl]amino]ethane sulfonic acid) was prepared from cholic acid. In the first step BA S1 was converted to the respective methyl ester utilizing thionyl chloride in methanol (Figure 2(Fig. 2)). Esterification was followed by selective mesylation of the hydroxy group in position 3 in 88 % yield. Selectivity is hereby presumed to be given due to an increased steric hindrance of the remaining hydroxy groups in position 7 and 12 as well as their axial orientation (Zhao and Zhong, 2005[70]). Conversion of mesylate S2 to the corresponding azide S3 by S_N_2 using sodium azide led to inversion of stereochemistry. Then, Staudinger reduction resulted in primary amine formation in an excellent yield. The following introduction of the chromophore was achieved by nucleophilic aromatic substitution (S_N_Ar) of 4-chloro-7-nitrobenzo-2-oxa-1,3-diazole (NBD) in methanol to give S5 with a 74 % yield. S_N_Ar was herein promoted by the electron withdrawing nitro group. Saponification followed by peptide coupling to the unmodified amino sulfonic acid taurine S8 using TBTU and HOBt led to the formation of S7. Unfortunately, purification of the respective taurocholate proved difficult due to its high polarity and water solubility. Hence, taurine was protected as the trifluoro ethanol sulfonic acid ester. Boc protection of the primary amine and chlorination followed by *in situ* esterification of the sulfonic acid utilizing trifluoro ethanol generated the protected taurine S10. The Boc protection group was then removed under acidic conditions. Peptide coupling of S11 to 3β-NBD cholic acid S5 and deprotection of the sulfonic acid ester then generated the desired product 3β-NBD-TCA S7 with an overall yield of 11 % over 8 steps.

### Transport experiments with 3β-NBD-TCA and [^3^H]-TCA in mAsbt-HEK and mNtcp-HEK cells

Qualitative transport experiments were performed in mNtcp-HEK and mAsbt-HEK cells with the fluorescent BA 4-nitrobenzo-2-oxa-1,3-diazole taurocholic acid (3β-NBD-TCA) over 10 min in sodium-containing transport buffer as previously reported (Lowjaga et al., 2021[40]). Fluorescence was captured by Leica DMI6000 B inverted fluorescent microscope at 10x magnification and analyzed by using the LAS X software (Leica, Wetzlar, Germany). Quantitative transport measurements were performed with 3β-NBD-TCA or [^3^H]-TCA in the mNtcp-HEK and mAsbt-HEK cells as reported before (Geyer et al., 2007[20]; Lowjaga et al., 2021[40]). Briefly, the cells were seeded into polylysine-coated 96-well plates and induced by tetracycline (1 µg/mL). After 72 h, growth medium was aspirated and each well was rinsed three times with 0.5 mL of incubation buffer (Hanks' balanced salt solution [HBSS] buffer supplemented with 20 mM HEPES, pH 7.4) and incubated for at least 20 min at 37 °C. The incubation buffer (further referred to as transport buffer) was removed and 200 µL of incubation buffer, containing 3β-NBD-TCA or [^3^H]-TCA, was added to each well and incubated at 37 °C for 10 min. After incubation, the uptake was terminated by aspirating the reaction mixture and washing the cells three times with 0.4 mL of ice-cold PBS. For the 3β-NBD-TCA, cell-associated fluorescence was directly measured by Glomax fluorescence reader (Promega) at 488 nm. In the case of [^3^H]-TCA, cells were solubilized with 0.6 mL of 1 N NaOH overnight. Cell-associated radioactivity of the [^3^H]-TCA was measured after the addition of 2.5 mL of Rotieco plus scintillation cocktail (Carl Roth, Karlsruhe, Germany) in a Tri-Carb 2910 TR scintillation counter (Perkin Elmer, Waltham, MA, USA).

### Mice and treatments

Male 8-10-week-old mOatp1a/1b cluster knockout mice, further referred to as Oatp^KO^ mice (Taconic Biosciences, USA; Cat. No. 10707-M; FVB.129P2-Del(*Slco1b2-Slco1a5*) 1Ahs) and corresponding wild-type mice (Taconic Biosciences, USA; Cat. No. FVB-M) were used. The mice were housed under standard conditions with free access to feed (Ssniff, Soest, Germany) and water. All experiments were approved by the local animal welfare committee (LANUV, North Rhine-Westphalia, Germany, application number: 84-02.04.2016.A279). Myrcludex B (5 mg/ kg) was administered intravenously 30 min before imaging. AS0369 (60 mg/kg) was administered orally by gavage 2 h before imaging.

### Intravital imaging

Functional intravital imaging of 3β-NBD-TCA transport in mouse liver and kidney was performed using a two-photon microscope (Zeiss, Germany) as previously described (Ghallab et al., 2024[21]). Tail vein bolus injections of the nuclear marker Hoechst 33258 and the mitochondrial membrane potential marker tetramethylrhodamine, ethyl ester (TMRE) was administered ~15 min before the start of recordings to visualize the liver and kidney morphology. To analyze the flux of 3β-NBD-TCA in the liver and kidneys, a bolus (50 µg/mouse for the liver experiments; 100 µg/mouse for the kidney experiments) was administered in the tail vein via a mouse catheter (SAI-infusion, IL, USA) a few seconds after the start of recordings. 

### Image analysis

As preprocessing for quantification of intravital imaging, rigid-body registration was performed using StackReg (Thévenaz et al., 1998[64]) to compensate for tissue motion (e.g., due to respiration and heartbeat) in the time series. Two-dimensional projections were created from these stabilized videos by z-projection using the average, sum, maximum, and standard deviation operators. The autocontext segmentation workflow of the ilastik interactive image segmentation software (version 1.3.3post1) (Berg et al., 2019[4]) was used to segment the tissue compartments in these 2D projections. Compartments considered for liver encompassed sinusoids, cells, and bile canaliculi and for the kidney included peritubular capillaries and TMRE+/- tubular cells. Mean raw 3β-NBD-TCA green intensities were measured per compartment and frame. Additionally, in kidney time series the mean 3β-NBD-TCA signal in the TMRE+ cell compartment was measured per tubule, and tubules were subsequently separated into two groups based on their maximum mean 3β-NBD-TCA intensity over time using k-means clustering.

### Immunohistochemistry

Triple co-staining of AQP1 (aquaporin-1; a marker of proximal TEC), TSC (thiazide sensitive NaCl cotransporter; a marker of distal tubules), and mAsbt was performed in 4 µm-thick PFA (4 %)-fixed paraffin-embedded kidney tissue sections using the Discovery Ultra Automated Slide Preparation System, as previously described (Ghallab et al., 2024[21]).

### Statistical analysis

Data were analyzed using GraphPad Prism 10.0.0 Software. Statistical analysis was done using Unpaired t test, as indicated in the figure legends.

## Results

### Synthesis and analysis of the transport properties of 3β-NBD-TCA

**Figure 3:**
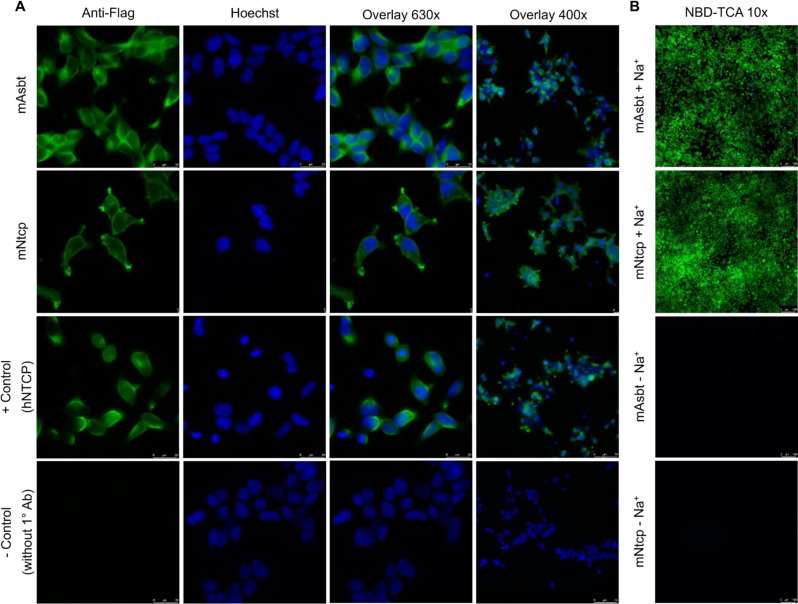
Expression of the mouse BA carriers mNtcp and mAsbt in HEK293 cells. A. Immunofluorescence microscopy of mAsbt-HEK and mNtcp-HEK cells. The images show anti-FLAG-fluorescence (green fluorescence). Nuclei were stained with Hoechst 33258 (blue fluorescence). HEK293 cells expressing the human Ntcp-FLAG protein were used as positive control and immunostaining without the anti-FLAG primary antibody was used as negative control. B. Expression of functionally active mNtcp and mAsbt carriers was verified by sodium-dependent uptake of 3β-NBD-TCA.

**Figure 4:**
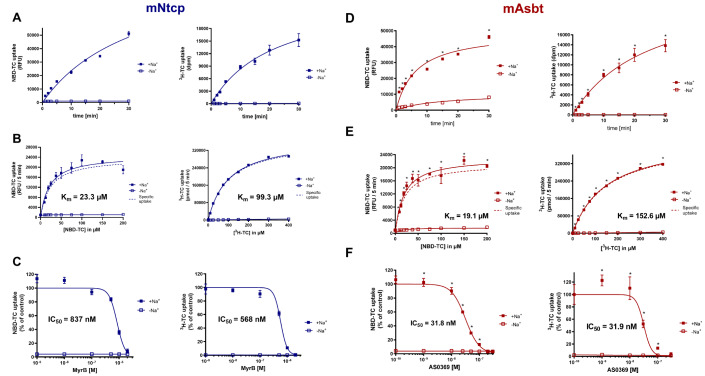
Transport kinetics of 3β-NBD-TCA compared to [^3^H]-TCA in mNtcp-HEK (A-C) and mAsbt-HEK (D-F) cells. (A, D) Time-dependent uptake in the presence (+Na^+^) and absence (-Na^+^) of sodium in the transport buffer up to 30 min. (B, E) Concentration-dependent uptake in the presence (+Na^+^) and absence (-Na^+^) of sodium in the transport buffer up to 50 µM. The carrier-specific uptake was calculated by subtracting the uptake under sodium-free conditions (dashed line). (C) Inhibition of the mNtcp-mediated BA transport by Myrcludex B (MyrB) and (F) inhibition of the mAsbt-mediated BA transport by AS0369 at increasing inhibitor concentrations. Half-maximal inhibitory concentrations (IC_50_) were calculated by non-linear regression analysis. Data represent means ± SD of quadruplicate determinations of representative experiments. *Significantly higher uptake compared to control; Unpaired t test, p < 0.0001

Several BA analogs have previously been developed where small fluorophores such as NBD or DBD ([1,3]dioxolo[4,5]benzodioxole) were coupled to the BA side chain (De Bruyn et al., 2014[11]; Leuenberger et al., 2021[38]; Maglova et al., 1995[41]). In the present study we coupled NBD at the 3b-position of taurocholic acid to generate 3β-NBD-TCA (Figure 2(Fig. 2)), a fluorescent BA that showed previously efficient hepatobiliary excretion in a rat *in situ* liver perfusion model, comparable to parent TCA (Petzinger et al., 1999[52]; Schramm et al., 1991[59]). Although present in all naturally occurring BAs, transport and pharmacophore modeling studies had shown that BA can be modified at the 3-hydroxy group without significantly impairing substrate recognition by BA transporters (Baringhaus et al., 1999[3]; Kramer et al., 1999[35]; Petzinger et al., 1999[52]). To address the question of how addition of an NBD-group at the 3-hydroxy position may affect the transport properties of TCA, we used HEK cells stably expressing mouse Ntcp (mNtcp) and ASBT (mAsbt) to compare the time- and concentration-dependent transport of 3β-NBD-TCA versus [^3^H]-TCA and the IC_50_ of specific BA transporter inhibitors (Figures 3(Fig. 3), 4(Fig. 4)). The FLAG-tagged mNtcp and mAsbt proteins were clearly detected in the plasma membrane as demonstrated with anti-FLAG immunofluorescence microscopy (Figure 3A(Fig. 3)). Both cell lines, mNtcp-HEK and mAsbt-HEK, showed sodium-dependent transport 3β-NBD-TCA (Figure 3B(Fig. 3)). The mNtcp-HEK cells showed nearly identical time-dependent (up to 30 min, Figure 4A(Fig. 4)) and comparable concentration-dependent uptake (Figure 4B(Fig. 4)) of 3β-NBD-TCA and [^3^H]-TCA. However, 3β-NBD-TCA was transported with higher affinity (K_m_ = 23.3 µM) compared to [^3^H]-TCA (K_m_ = 99.3 µM) (Figure 4B(Fig. 4)). The transport of both substrates was strictly sodium-dependent (Figure 4A; 4B(Fig. 4)). Using the Ntcp inhibitor Myrcludex B, an IC_50_ of 837 nM was obtained for 3β-NBD-TCA, compared to 568 nM for [^3^H]-TCA (Figure 4C(Fig. 4)). HEK293 cells stably expressing mAsbt showed similar time-dependent (up to 30 min; Figure 4D(Fig. 4)) and concentration-dependent (Figure 4E(Fig. 4)) uptake of NBC-TCA compared to [^3^H]-TCA. However, compared to the mNtcp-HEK cells, there seemed to be a somewhat higher unspecific uptake of 3β-NBD-TCA in the absence of sodium (Figure 4D, 4E(Fig. 4) vs. Figure 4A, 4B(Fig. 4)) in the mAsbt-HEK cells. Of note, there was strictly no uptake of [^3^H]-TCA in the mAsbt-HEK cells in the sodium-free buffer. As for mNtcp, 3β-NBD-TCA showed higher affinity transport (K_m_ = 19.1 µM) compared to [^3^H]-TCA (K_m_ = 152.6 µM) (Figure 4B(Fig. 4)). Regarding transport inhibition, IC_50_ values for the mAsbt inhibitor AS0369 were almost identical regardless of the bile acid used (Figure 4F(Fig. 4)). Thus, no major differences in uptake and inhibition kinetics by mNtcp and mAsbt were observed *in vitro* due to the attachment of the NBD-fluorophore to the TCA molecule using [^3^H]-TCA as the reference. Therefore, 3β-NBD-TCA was further validated by intravital imaging in WT mice and in mouse models using genetic or pharmacologic approaches to selectively inhibit BA uptake transporters. 

### Mouse Oatp1a/1b and Ntcp are responsible for 3β-NBD-TCA uptake from sinusoidal blood into hepatocytes

**Figure 5:**
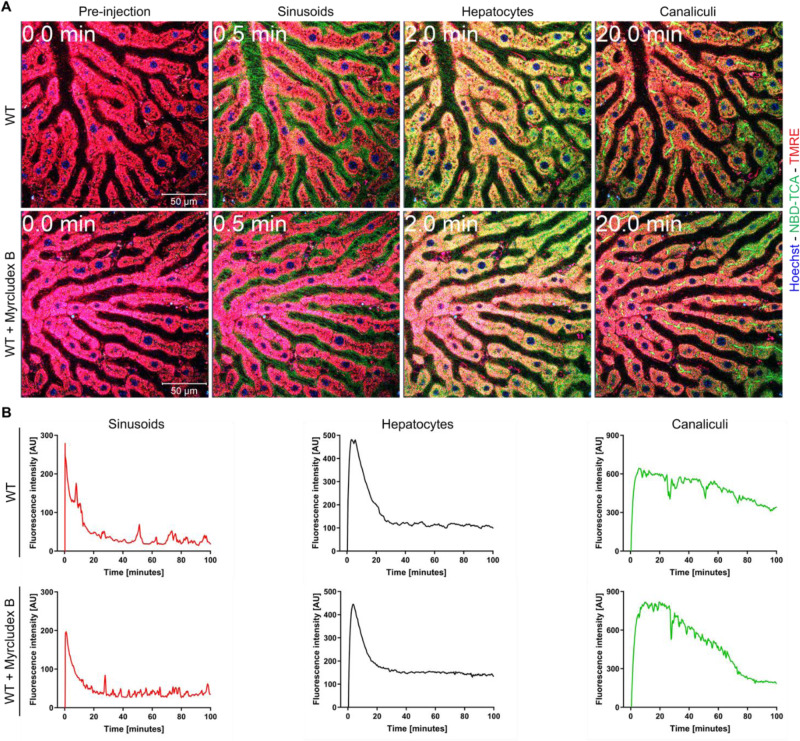
Kinetics of 3β-NBD-TCA in livers of WT mice with and without administration of Myrcludex B. A. Stills from 2-photon videos. B. Quantification of 3β-NBD-TCA-associated fluorescence in sinusoids, hepatocytes, and canaliculi of segmented videos. Red: TMRE; green: 3β-NBD-TCA; blue: Hoechst 33258. The data corresponds to supplementary videos 1 and 2. The data are from a representative mouse out of 3 mice per condition.

**Figure 6:**
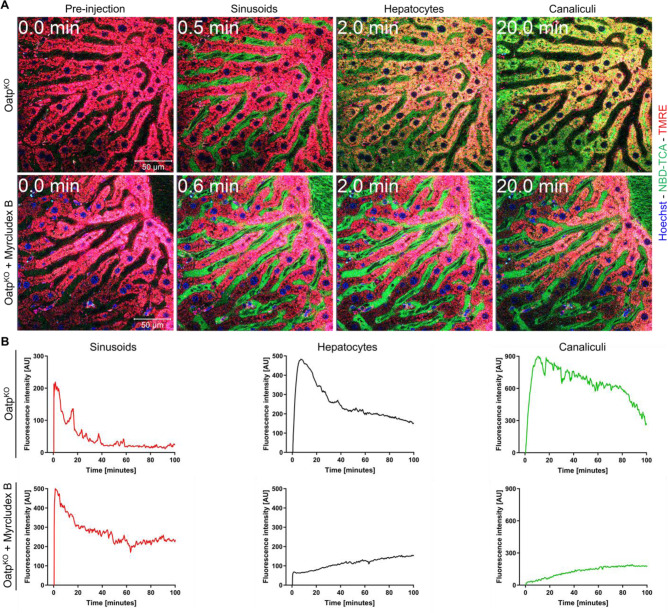
Kinetics of 3β-NBD-TCA in livers of Oatp^KO^ mice with and without Myrcludex B administration. A. Stills from 2-photon videos. B. Quantification of 3β-NBD-TCA-associated fluorescence in sinusoids, hepatocytes, and canaliculi of segmented videos. Red: TMRE; green: 3β-NBD-TCA; blue: Hoechst 33258. The data corresponds to supplementary videos 3 and 4. The data are from a representative mouse out of 3 mice per condition.

*In vivo* kinetics of 3β-NBD-TCA flux in the liver sinusoids (blood), hepatocytes and bile canaliculi were analyzed by intravital imaging after tail vein bolus injection and were quantified using segmented 2-photon videos (Figures 5(Fig. 5), 6(Fig. 6)). In wild-type (WT) mice, 3β-NBD-TCA associated green fluorescence appeared in the sinusoidal blood within seconds after injection, followed by uptake into hepatocytes and secretion into bile canaliculi (Figure 5A(Fig. 5); Suppl. video 1, see also the legends to supplementary videos). Administration of the Ntcp inhibitor Myrcludex B (5 mg/kg) 30 min before 3β-NBD-TCA injection did not lead to a visible change in hepatocellular uptake or canalicular secretion of this BA (Figure 5A(Fig. 5); Suppl. video 2). Quantification of fluorescence intensity in videos with segmented sinusoids, hepatocytes and bile canaliculi confirmed that administration of the Ntcp inhibitor did not alter the clearance of 3β-NBD-TCA from sinusoidal blood or the kinetics of fluorophore appearance in hepatocytes (Figure 5B(Fig. 5)). 

Since TCA has been shown to be transported by both mNtcp and organic anion transporting polypeptide subfamily mOatp1a/ 1b carriers in WT mice (Slijepcevic et al., 2017[61]), we repeated the intravital imaging in Oatp^KO^ mice lacking the entire *Slco1b2-Slco1a5 *gene cluster. Administration of Myrcludex B induced a massive change in hepatocellular clearance for 3β-NBD-TCA in the Oatp^KO^ mice (Figure 6(Fig. 6); Suppl. videos 3 and 4). In Oatp^KO^ mice, clearance of 3β-NBD-TCA from blood into hepatocytes was almost completely blocked by Myrcludex B. Nevertheless, the very small amount of 3β-NBD-TCA that entered the hepatocytes was rapidly secreted into the bile canaliculi. These experiments confirm previous results (Slijepcevic et al., 2017[61]) and demonstrate that both mNtcp and mOatp1a/1b carriers contribute to 3β-NBD-TCA uptake from sinusoidal blood into hepatocytes and are able to compensate for each other under these *in vivo* conditions. 

### Inhibition of mNtcp in Oatp1a/1b knockout mice causes an increase of 3β-NBD-TCA in renal capillaries and proximal TEC

**Figure 7:**
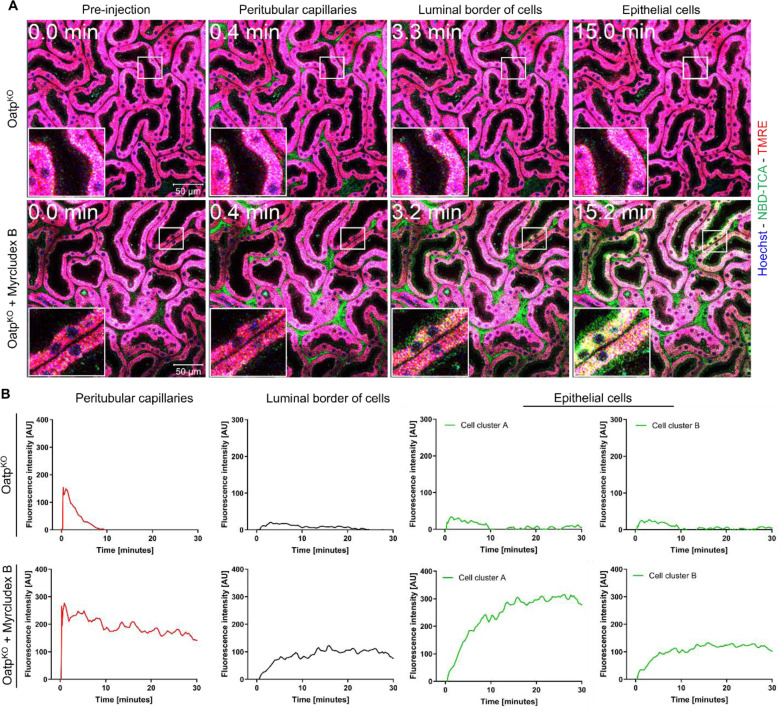
Kinetics of 3β-NBD-TCA in kidneys of Oatp^KO^ mice with and without Myrcludex B administration. A. Stills from 2-photon videos. B. Quantification of 3β-NBD-TCA-associated fluorescence in renal peritubular capillaries, tubular lumen, and tubular epithelial cells. Red: TMRE; green: 3β-NBD-TCA; blue: Hoechst 33258. The data corresponds to supplementary videos 5 and 6. The data are from a representative mouse out of 3 mice per condition.

Recently, it has been shown that cholestasis induced by bile duct ligation (BDL) in mice leads to increased concentrations of BAs in renal capillaries, enhanced glomerular filtration, increased BA concentration in renal tubules and increased mAsbt-driven uptake of BA into proximal TEC (Ghallab et al., 2024[21]). To study if this can also be observed in the absence of liver disease in mice, we performed intravital imaging of the kidneys of Oatp^KO^ mice treated with Myrcludex B after tail vein bolus injection of 3β-NBD-TCA and quantified fluorescence in renal capillaries and tubular epithelial cells using segmented 2-photon videos (Figure 7(Fig. 7); Suppl. videos 5 and 6). Without administration of Myrcludex B, Oatp^KO^ mice exhibited only a short transient increase of fluorescence in the renal peritubular capillaries (Suppl. video 5; Figure 7(Fig. 7)). In contrast, Oatp^KO^ mice treated with Myrcludex B exhibited a strong and persistent 3β-NBD-TCA signal in renal peritubular capillaries (Suppl. video 6; Figure 7(Fig. 7)). The increased green-fluorescent signal in renal peritubular capillaries is accompanied by the uptake of 3β-NBD-TCA into some TEC (Figure 7A(Fig. 7); Suppl. video 6) occurring as yellow fluorescence in the images due to red TMRE background fluorescence. Upon close inspection of Suppl. video 6 and Figure 7A(Fig. 7) (with Myrcludex B), 3β-NBD-TCA was initially visualized on the extracellular luminal side of TEC, which was followed by enrichment intracellularly in some of the red TMRE-positive cells (Figure 7A, B(Fig. 7); Suppl. video 6). This extracellular luminal enrichment was observed in both the proximal and distal renal tubular epithelial cells. Taken together, blocking of the hepatic BA uptake transporters causes alternative renal filtration of 3β-NBD-TCA and zonated enrichment in some TEC. 

### AS0369 inhibits 3β-NBD-TCA enrichment in proximal TEC

**Figure 8:**
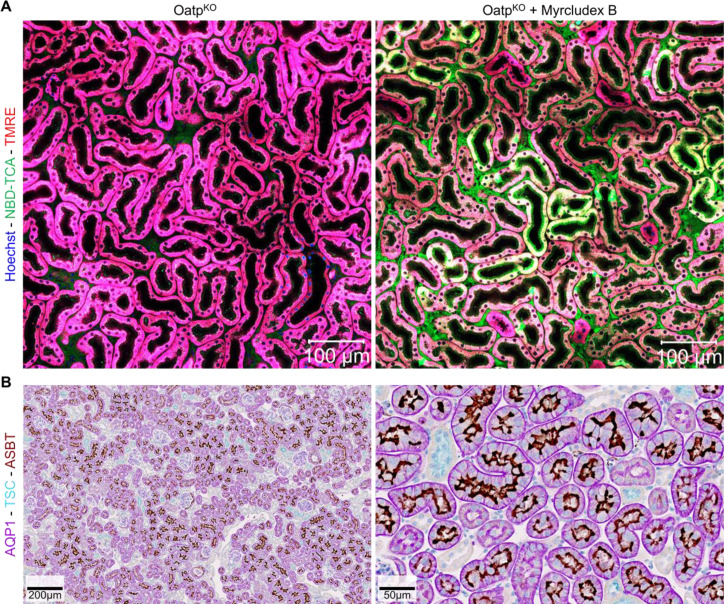
Zonated enrichment of 3β-NBD-TCA in a subpopulation of renal proximal TEC in Oatp^KO^ mice injected with Myrcludex B. A. Stills before and 30 min after injection of 3β-NBD-TCA. B. Co-immunostaining of the kidneys of Oatp^KO^ mice for aquaporin1 (AQP1), thiazide sensitive NaCl cotransporter (TSC) and mAsbt. The dark mAsbt-associated staining is seen on the luminal membrane of a subpopulation of AQP1 positive TEC. The data are from a representative mouse out of 3 mice per condition.

In addition to glomerular filtration, it has been reported that BAs may be excreted in urine by tubular secretion by proximal tubule cells. This raises the question of whether the accumulation of 3β-NBD-TCA in proximal tubule cells under these conditions is primarily due to ASBT-mediated absorption or due to other mechanisms such as OAT3-mediated uptake from the blood. For this purpose, we first analyzed mAsbt expression in the Oatp^KO^ mice. Similar to the pattern for 3β-NBD-TCA enrichment of TEC (Figure 8A(Fig. 8)), a subpopulation of TEC expressed mAsbt at their luminal side (Figure 8B(Fig. 8)). Co-immunostaining of the proximal TEC marker aquaporin 1 (AQP1) and mAsbt demonstrated that mAsbt was expressed exclusively in AQP1-positive TEC (Figure 8B(Fig. 8)). It should also be noted that not all AQP1-positive TEC were mAsbt positive; thus, proximal TEC can be subdivided into mAsbt positive and negative subpopulations. 

**Figure 9:**
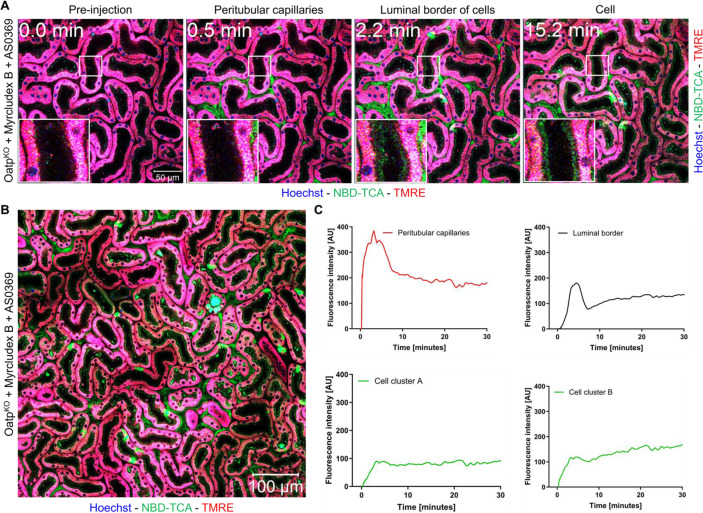
Inhibition of mAsbt by AS0369 blocks 3β-NBD-TCA enrichment in renal proximal TEC of Oatp^KO^ mice treated with Myrcludex B. A. Stills of 2-photon videos. B. Overview image recorded 30 min after 3β-NBD-TCA administration. C. Quantification of 3β-NBD-TCA-associated fluorescence in renal peritubular capillaries, tubular lumen, and tubular epithelial cells. Red: TMRE; green: 3β-NBD-TCA; blue: Hoechst 33258. The data corresponds to supplementary video 7. The data are from a representative mouse out of 3 mice.

To functionally characterize the mAsbt positive TEC, Myrcludex B-treated Oatp^KO^ mice received 60 mg/kg AS0369 orally by gavage 2 h before tail vein injection of 3β-NBD-TCA (Figure 9(Fig. 9), Suppl. video 7). In contrast to vehicle controls, AS0369 pre-injected mice showed strongly reduced enrichment of 3β-NBD-TCA in TEC. It should be noted that the strong and sustained 3β-NBD-TCA signal in renal capillaries was not influenced by AS0369 pre-injection (Figure 9(Fig. 9)). Interestingly, the luminal border of all TEC showed the green-fluorescent 3β-NBD-TCA signal shortly after administration but the uptake into the cells (TMRE positive) was blocked (Figure 9A-C(Fig. 9)) after ASBT inhibition with AS0369. 

## Discussion

In the present study, we validated NBD-coupled taurocholic acid (3β-NBD-TCA) for intravital imaging of hepatorenal transport of BA. *In vitro*, similar transport properties of 3β-NBD-TCA were observed for mNtcp and mAsbt compared to a radioactively labeled [^3^H]-TCA, demonstrating that the fluorophore did not alter transport kinetics to a relevant extent. For mNtcp expressed in HEK293 cells, a K_m_ value of 99.3 µM was measured for [^3^H]-TCA in the present studies, comparable to a previous study on mNtcp expressed in *Xenopus laevis* oocytes (K_m_ = 86 µM) (Cattori et al., 1999[8]). This high K_m_ value can be explained by the high-capacity transport of [^3^H]-TCA in the mNtcp-HEK and in the mAsbt-HEK cells that was not yet completely saturated at very high substrate concentrations of 400 µM. In contrast, 3β-NBD-TCA showed saturated transport kinetics at substrate concentrations up to 200 µM and lower K_m_ values for mNtcp and mAsbt of 23.3 µM and 19.1 µM, respectively. Of note, up to a substrate concentration of 50 µM that is considered the relevant concentration range in the *in vivo* studies, concentration-dependent transport of [^3^H]-TCA and 3β-NBD-TCA was nearly identical. *In vivo *in mice, only a combination of both, knockout of the hepatic mOatp1a/1b carriers in OATP^KO^ mice plus pharmacological inhibition of mNtcp completely blocked the uptake of 3β-NBD-TCA from blood into hepatocytes. Inhibition of mNtcp in WT mice or knockout of the mOtap1a/1b carriers without mNtcp inhibition did not lead to relevantly altered kinetics. This observation confirmed the data of a previous study (Slijepcevic et al., 2017[61]). An interesting finding was that the increase of 3β-NBD-TCA in the systemic circulation after combined mOatp1a/1b deletion and mNtcp inhibition led to enhanced glomerular filtration and to 3β-NBD-TCA enrichment in a subpopulation of proximal TEC. The enrichment of the fluorophore-coupled TCA was almost completely blocked by the systemically available ASBT inhibitor AS0369. These results align with a previous study, where cholestasis was induced by ligation of the common bile duct (Ghallab et al., 2024[21]) and the subsequent bile acid uptake and damage was prevented by AS0369. 

An unexpected observation was the enrichment of 3β-NBD-TCA at the luminal side of both proximal and distal TEC. This enrichment did not overlap with the region of mitochondrial staining by TMRE, suggesting that 3β-NBD-TCA bound to extracellular structures. This would agree with the observation that the luminal enrichment of 3β-NBD-TCA was also seen under conditions of mAsbt inhibition with compound AS0369, which efficiently prevented uptake of 3β-NBD-TCA into the cytoplasm of proximal TEC. A possible interpretation may be that BA are bound by extracellular structures attached to the luminal membrane of TEC (both proximal and distal). This may lead to increased local BA enrichment so that mAsbt can work under conditions of high substrate concentrations. The site of accumulation of 3β-NBD-TCA at the extracellular luminal space of TEC should not be misinterpreted as the brush-border, which represents the microvillus-covered surface of the epithelial cells and is only observed for proximal (and not distal) TEC. In contrast, the enrichment of 3β-NBD-TCA at the luminal side was observed for both proximal and distal tubules and must, therefore, be due to a mechanism which exists in all (proximal and distal) TEC. 

Cholestatic liver diseases require effective therapies to reduce the increased burden of death and liver transplantation (Beuers et al., 2015[5]; Fuchs and Trauner, 2022[17]; Trauner and Fuchs, 2022[65]). An important component in the development of new therapies are animal models of cholestasis (Mariotti et al., 2019[43]; Perlman, 2016[51]; Rosenthal and Brown, 2007[57]). Among the existing models, bile duct ligation (BDL) has been the first and probably most extensively used technique (Caballero-Camino et al., 2023[7]; Gee et al., 2023[18]; Ghallab et al., 2019[24]; Tag et al., 2015[63]). Other genetically modified mouse strains are also available, such as the Mdr2^-/-^ (*Abcb4^-/-^*) mice that have been used as a model of sclerosing cholangitis (Fickert and Wagner, 2017[16]; Moncsek et al., 2018[46]) or genetic mouse models of Alagille syndrome (Adams et al., 2020[1]; Hankeova et al., 2021[27]; Niknejad et al., 2023[47]). Moreover, several progressive cholestatic animal models induced by toxicants are used, such as 3,5‐diethoxycarbonyl‐1,4‐dihydrocollidine (DDC) (Slijepcevic et al., 2017[61]; Wang et al., 2022[67]) or alpha-naphthyl isothiocyanate (ANIT) (Greenman et al., 2024[26]; Santamaría et al., 2019[58]). These models have in common that besides increasing BA concentrations in the systemic circulation, they also damage liver tissue (Ghallab et al., 2019[24]). For example, damage associated molecular patterns (DAMPs) (Hao et al. 2017[28]; Woolbright et al., 2013[69]) and cytokines (Ommati et al., 2023[48]; Peña-Rodríguez et al., 2022[50]) are released in response to injury and may contribute to the liver disease. For several non-hepatic complications of cholestatic liver disease, for example cholemic nephropathy- a severe and often fatal complication of several liver diseases with an unmet need for therapy (Krones et al., 2018[36]; Mandorfer et al., 2023[42]; Simbrunner et al., 2021[60]) - the specific contribution of different mediators released from the cholestatic liver, such as BA, DAMPs or cytokines still needs to be clarified (Fickert, 2024[14]; Fickert and Rosenkranz, 2020[15]; Krones et al., 2018[36]). To develop optimal targeted therapeutic strategies, it is important to know if BA alone are responsible for the systemic adverse effects or if BA synergize with other inflammatory mediators. If BA represent the only pathophysiological principle, treatment with a systemic ASBT inhibitor that causes urinary excretion and systemic reduction of BA (Ghallab et al., 2024[21]) should be sufficient; if inflammatory mediators contribute, the ASBT inhibitor should be combined with anti-inflammatory drugs. The here validated probe for spatio-temporal intravital analysis of BA transport offers excellent technical possibilities to understand hepato-renal BA homeostasis in health and disease. Moreover, the mouse model used in the present study, namely Myrcludex B-treated Oatp^KO^ mice (briefly referred to as Oatp-Ntcp-model) represents a suitable genetic/pharmacological tool to study the specific contribution of BA to systemic complications of cholestatic liver diseases. The intervention is well-defined, since the substrate specificity of the BA transporters mNtcp and mOatp1a/1b in mice is known (Geier et al., 2003[19]; Orozco et al., 2023[49]) and the deletion of mOatp1a/1b together with the inhibition of mNtcp leads to an increase of a specific spectrum of BA (Donkers et al., 2020[13]; Slijepcevic et al., 2017[61]). Importantly, the required intervention (the injection of Myrcludex B) does not induce damage to liver tissue so that the Oatp-Ntcp model allows the specific analysis of the consequences of increased conjugated BA, without the additional influence of DAMPs and cytokines. In contrast, the integrated effect of all cholephiles (e.g., BA plus DAMPs plus cytokines) can be studied with the BDL mouse model. It should also be considered that the required injection of Myrcludex B in the Oatp-Ntcp mouse model induces much less pain, suffering and damage than the surgical intervention required for BDL, long-term administration of toxicants, or the breeding of genetically modified animals with a disease phenotype, such as Mdr2-/- mice. Importantly, the here applied OATP^KO^ mice do not show any spontaneous adverse health effects, since the loss of the mOatp1a/1b function regarding hepatic BA transport is compensated by mNtcp. Although not yet studied, this model allows for studies of long-term pharmacological inhibition of mNtcp in OATP^KO^ mice and the secondary consequences of long-term elevations of serum BA. However, for mechanistic studies the here applied short term experiment is sufficient, since, e.g., the intravitally detectable accumulation of BA in TEC - an early critical mechanism in the pathophysiology of cholemic nephropathy (Ghallab et al., 2024[21]) - is already active immediately after mNtcp inhibition. Thus, therapeutic interventions, such as transporter inhibition and antioxidative agents can already be studied in short-term experiments. 

It should be considered that the hepatic BA transport is different between mice and human. While Ntcp inhibition or knockout in mice seems to be compensated by increased BA uptake via the hepatic mOatps, humans with genetic Ntcp deficiency show remarkable and persistent hyperchloremia at least in younger patients (Qiu et al., 2017[53]). In addition, Myrcludex B treatment in humans leads to increased serum BA levels, indicating that the human OATPs are not efficient in hepatic BA uptake to compensate for Ntcp deficiency/inhibition (Blank et al., 2016[6]). The mouse/human hepatic Oatps/OATPs from the 1a/1b subfamilies are different. In human liver, OATP1B1, OATP1B3 are expressed (Kopplow et al., 2005[34]), in the mouse liver Oatp1a1, 1a4, and 1b2 are expressed (van de Steeg et al., 2010[66]). 

A limitation of the here presented Oatp-Ntcp mouse model is that besides BA plasma bilirubin is also increased due to deletion of the hepatic mOatp1a/1b carriers that are involved in hepatic bilirubin uptake (Li et al., 2023[39]; van de Steeg et al., 2010[66]). Therefore, additional efforts would be needed, when it is important to differentiate between BA- and bilirubin-mediated pathomechanisms. In this case OATP^KO^ mice are important as additional control that show increased plasma bilirubin without enhancing BA levels (Li et al., 2023[39]). 

In conclusion, the Oatp-Ntcp mouse model allows the analysis of pathomechanisms due to increased BA concentrations in the systemic blood without the additional influence of DAMPs and cytokines. In this model the here validated fluorescent 3β-NBD-TCA BA derivative represents a valuable surrogate molecule to visualize the hepatobiliary as well as the renal proximal tubule BA fluxes by intravital imaging.

## Notes

Jan G. Hengstler and Joachim Geyer contributed equally as senior author.

Ahmed Ghallab, Jan G. Hengstler (Department of Toxicology, Leibniz Research Centre for Working Environment and Human Factors, Technical University Dortmund, Ardeystr. 67, 44139 Dortmund, Germany; E-mail: hengstler@ifado.de) and Joachim Geyer (Institute of Pharmacology and Toxicology, Justus Liebig University Giessen, Biomedical Research Center Seltersberg, Schubertstr. 81, 35392 Giessen, Germany; E-mail: Joachim.M.Geyer@vetmed.uni-giessen.de) contributed equally as corresponding author.

## Declaration

### Funding statement

The study was supported in part by the Deutsche Forschungsgemeinschaft (DFG) SFB 1021, project number 197785619 (project B8 to J.G.), and project numbers 517010379& 457840828 (to A.G.). 

### Acknowledgment 

We thank Anita Neubauer and Bärbel Fühler- Institute of Pharmacology and Toxicology, Justus Liebig University Giessen, Biomedical Research Center Seltersberg, Schubertstr. 81, 35392 Giessen, Germany- for excellent technical assistance.

### Data availability statement

The data presented in this study are available on request from the corresponding author.

### Conflict of interest disclosure

Nothing to disclose.

## Supplementary Material

Supplementary information

Suppl. video 1

Legends to supplementary videos

Suppl. video 2

Suppl. video 3

Suppl. video 4

Suppl. video 5

Suppl. video 6

Suppl. video 7

## Figures and Tables

**Figure 1:**
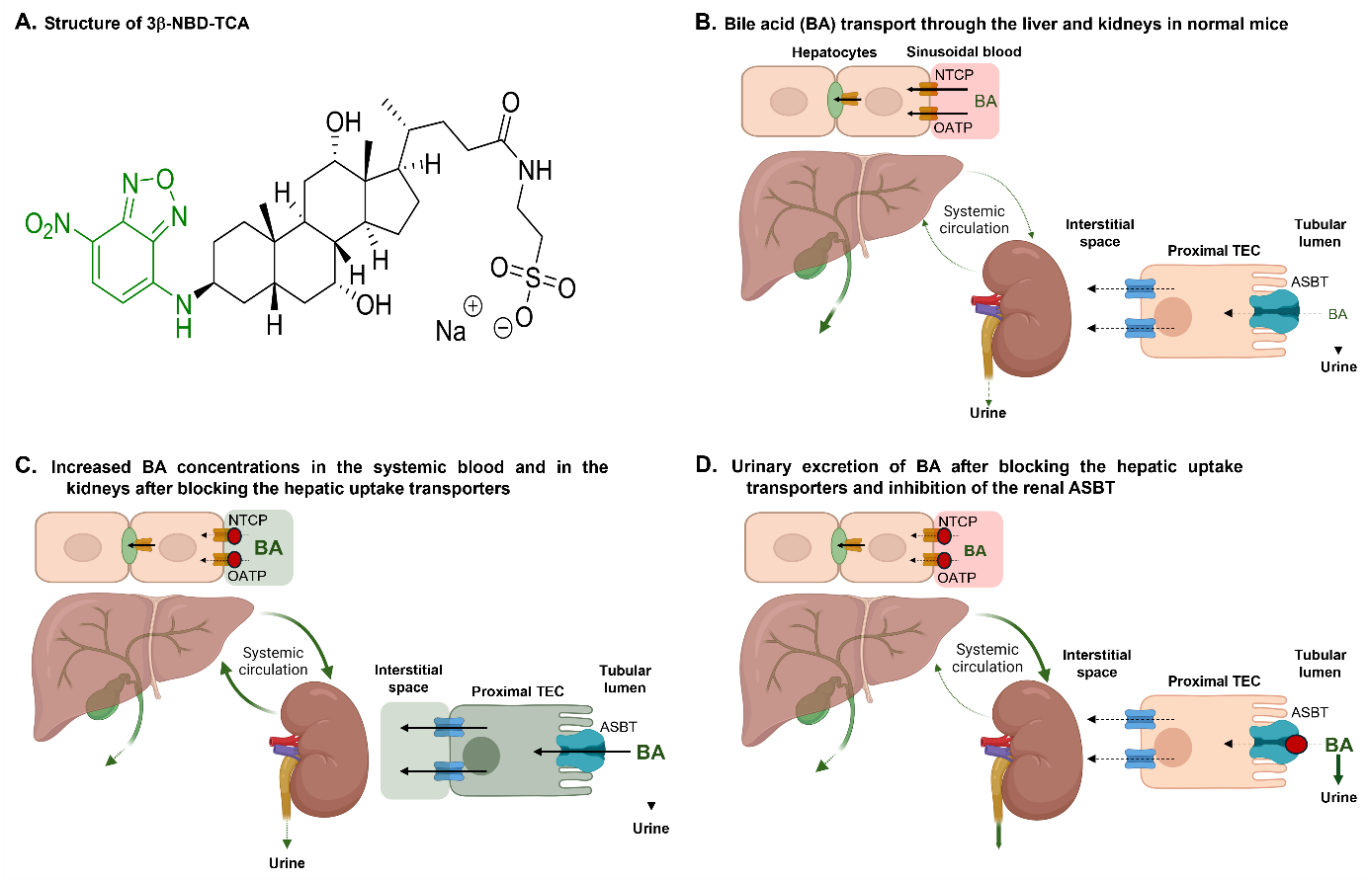
Graphical abstract
