# Supplementary material for: Validation of NBD-coupled taurocholic acid for intravital analysis of bile acid transport in liver and kidney of mice
Source: EXCLI J. 2024 Oct 30;23:1330–52. doi: 10.17179/excli2024-7707 (PMC11579514; doi:10.17179/excli2024-7707)
Supplement: Supplementary information [file EXCLI-23-1330-s-001.pdf]

EXCLI Journal 2024;23:1330-1352 – ISSN 1611-2156
Received: August 01, 2024, accepted: October 28, 2024, published: October 30, 2024

Supplementary information to:

Original article:

VALIDATION OF NBD-COUPLED TAUROCHOLIC ACID FOR INTRAVITAL ANALYSIS OF BILE ACID TRANSPORT IN LIVER AND KIDNEY OF MICE

Ahmed Ghallab[1,2*], Sebastian Kunz[3], Celine Drossel[4], Veronica Billo[3], Adrian Friebel[5], Mats Georg[4], Richard Göttlich[4], Zaynab Hobloss[1], Reham Hassan[1,2], Maiju Myllys[1], Abdel-latief Seddek[2], Noha Abdelmageed[6], Paul A. Dawson[7], Erik Lindström[8], Stefan Hoehme[5], Jan G. Hengstler[1*#], Joachim Geyer[3*#]

[1] Department of Toxicology, Leibniz Research Centre for Working Environment and Human Factors, Technical University Dortmund, Ardeystr. 67, 44139 Dortmund, Germany
[2] Department of Forensic Medicine and Toxicology, Faculty of Veterinary Medicine, South Valley University, 83523 Qena, Egypt
[3] Institute of Pharmacology and Toxicology, Justus Liebig University Giessen, Biomedical Research Center Seltersberg, Schubertstr. 81, 35392 Giessen, Germany
[4] Institute of Organic Chemistry, Justus Liebig University Giessen, Heinrich-Buff-Ring 17, 35392, Giessen, Germany
[5] Institute of Computer Science & Saxonian Incubator for Clinical Research (SIKT), University of Leipzig, Haertelstraße 16-18, 04107 Leipzig, Germany
[6] Department of Pharmacology, Faculty of Veterinary Medicine, Sohag University, 82524 Sohag, Egypt
[7] Department of Pediatrics, Division of Gastroenterology, Hepatology, and Nutrition, Emory University, Atlanta, GA 30322, USA
[8] Albireo Pharma, Inc., Boston, MA 02109, USA

[#] Indicates shared senior authorship
* **Corresponding authors:** Ahmed Ghallab, Jan G. Hengstler, Department of Toxicology, Leibniz Research Centre for Working Environment and Human Factors, Technical University Dortmund, Ardeystr. 67, 44139 Dortmund, Germany. E-mail: ghallab@ifado.de; hengstler@ifado.de
Joachim Geyer, Institute of Pharmacology and Toxicology, Justus Liebig University Giessen, Biomedical Research Center Seltersberg, Schubertstr. 81, 35392 Giessen, Germany. E-mail: Joachim.M.Geyer@vetmed.uni-giessen.de

https://dx.doi.org/10.17179/excli2024-7707

This is an Open Access article distributed under the terms of the Creative Commons Attribution License (http://creativecommons.org/licenses/by/4.0/).

EXCLI Journal 2024;23:1330-1352 – ISSN 1611-2156
Received: August 01, 2024, accepted: October 28, 2024, published: October 30, 2024

SUPPLEMENTAL EXPERIMENTAL PROCEDURES

Methyl-3α,7α,12α-trihydroxy-5β-cholan-24-oate

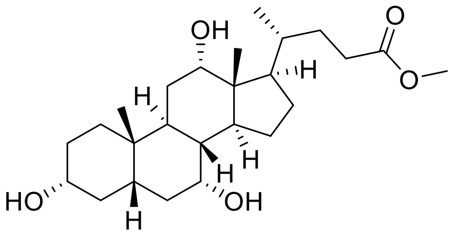

Under nitrogen atmosphere cholic acid (5.903 g, 14.447 mmol, 1 equiv.) was dissolved in 70 mL anhydrous methanol. Thionyl chloride (1.15 mL, 15.85 mmol, 1.1 equiv.) was added dropwise at 0 °C and the mixture was stirred for 18 h at room temperature (RT). The solvent was removed under reduced pressure. The resulting crude product was dissolved in 30 mL ethyl acetate and was washed with 30 mL saturated sodium bicarbonate, followed by 30 mL brine. The organic layer was dried over $MgSO_4$, and the solvent was removed under reduced pressure to obtain the product as a white solid (5.801 g, 13.727 mmol, 95 %).
HRMS (ESI): m/z = 445.2918 $[M+Na]^+$ (calculated for 445.2924)
^{1}H-NMR ($CDCl_3$, 400.1 MHz): δ [ppm] = 3.98-3.94 (m, 1H), 3.89-3.79 (m, 1H), 3.65 (s, 3H), 3.53-3.37 (m, 1H), 2.75 (s, 3H), 2.43-2.30 (m, 1H), 2.30-2.14 (m, 3H), 1.99-1.02 (m, 20H), 0.97 (d, *J* = 6.1 Hz, 3H), 0.88 (s, 3H), 0.67 (s, 3H).
^{13}C-NMR ($CDCl_3$, 100.6 MHz): δ [ppm] = 174.94, 73.20, 72.14, 68.60, 51.64, 47.18, 46.59, 41.86, 41.60, 39.62, 35.39, 34.88, 34.76, 31.23, 31.04, 30.52, 28.35, 27.62, 26.58, 23.24, 22.61, 17.45, 12.62.

Methyl-7α,12α-dihydroxy-3α-[(methylsulfonyl)oxy]-5β-cholan-24-oate S2

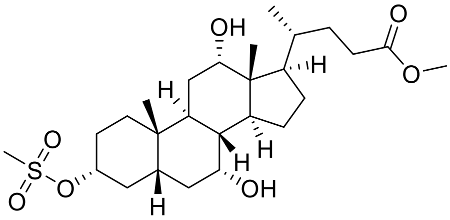

Under nitrogen atmosphere triethylamine (0.92 mL, 6.64 mmol, 2 equiv.) was added to a solution of methyl-3α,7α,12α-trihydroxy-5β-cholan-24-oate (1.405 g, 3.325 mmol, 1 equiv.) in 30 mL anhydrous dichloromethane. Methane sulfonyl chloride (0.26 mL, 3.35 mmol, 1 equiv.) in 5 mL anhydrous dichloromethane was then added to the mixture dropwise at 0 °C. The mixture was stirred for 2 h at 0 °C and was then quenched by the addition of 30 mL distilled water. The phases were separated and the aqueous layer was extracted three times with 30 mL dichloromethane. The combined organic layers were washed with 20 mL saturated sodium bicarbonate, distilled water and brine and then dried over $MgSO_4$. The solvent was removed under reduced pressure and the crude product was purified by flash column chromatography (ethyl acetate/cyclohexane 1:1). The product was obtained as a white foam (1.466 g, 2.928 mmol, 88 %).
HRMS (ESI): m/z = 523.2695 $[M+Na]^+$ (calculated for 523.2700)
^{1}H-NMR ($CDCl_3$, 400.1 MHz): δ [ppm] = 4.57-4.46 (m, 1H), 4.02-3.96 (m, 1H), 3.90-3.81 (m, 1H), 3.66 (s, 3H), 2.98 (s, 3H), 2.60 (q, *J* = 12.9 Hz, 1H), 2.44-2.31 (m, 1H), 2.28-2.09 (m, 2H), 2.02-1.23 (m, 21H), 1.22-1.10 (m, 1H), 0.97 (d, *J* = 6.2 Hz, 3H), 0.90 (s, 3H), 0.69 (s, 3H).
^{13}C-NMR ($CDCl_3$, 100.6 MHz): δ [ppm] = 173.70, 81.65, 71.79, 67.09, 50.53, 46.21, 45.52, 40.93, 40.41, 38.50, 37.92, 35.06, 34.13, 33.79, 33.46, 33.12, 30.03, 29.84, 27.31, 26.90, 26.41, 25.65, 22.11, 21.33, 16.34, 11.54.

EXCLI Journal 2024;23:1330-1352 – ISSN 1611-2156
Received: August 01, 2024, accepted: October 28, 2024, published: October 30, 2024

Methyl-3β-azido-7α,12α-dihydroxy-5β-cholan-24-oate S3

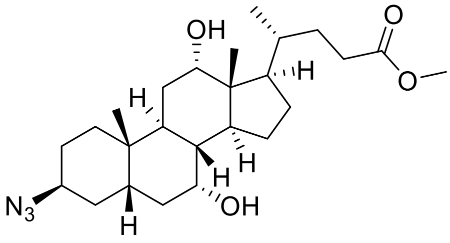

Under nitrogen atmosphere methyl-7α,12α-dihydroxy-3α-[(methylsulfonyl)oxy]-5β-cholan-24-oate (0.577 g, 1.152 mmol, 1 equiv.) and sodium azide (0.374 g, 5.760 mmol, 5 equiv.) were dissolved in 10 mL anhydrous dimethylformamide. The mixture was stirred for 48 h at 80 °C. Then 10 mL distilled water and 10 mL ethyl acetate were added to the mixture and the phases were separated. The aqueous layer was extracted three times with 20 mL ethyl acetate. The combined organic layers were washed three times with 20 mL brine and dried over $MgSO_4$. The solvent was removed under reduced pressure and the crude product was purified by flash column chromatography (ethyl acetate/ cyclohexane 1:1) to obtain the product as a white solid (0.399 g, 9.891 mmol, 77 %).
HRMS (ESI): m/z = 470.2991 $[M+Na]^+$ (calculated for 470.2989)
^{1}H-NMR ($CDCl_3$, 400.1 MHz): δ [ppm] = 4.00-3.95 (m, 1H), 3.92-3.88 (m, 1H), 3.88-3.83 (m, 1H), 3.66 (s, 3H), 2.58-2.48 (m, 1H), 2.40-2.31 (m, 1H), 2.27-2.08 (m, 2H), 2.02-1.26 (m, 21H), 1.19-1.10 (m, 1H), 0.97 (d, *J* = 6.3 Hz, 3H), 0.92 (s, 3H), 0.69 (s, 3H).
^{13}C-NMR ($CDCl_3$, 100.6 MHz): δ [ppm] = 174.83, 73.09, 68.54, 58.85, 51.67, 47.41, 46.71, 42.13, 39.61, 36.90, 35.29, 35.21, 34.20, 33.17, 31.20, 30.99, 30.63, 28.64, 27.58, 26.38, 24.70, 23.31, 23.03, 17.48, 12.68.

Methyl-3β-amino-7α,12α-dihydroxy-5β-cholan-24-oate S4

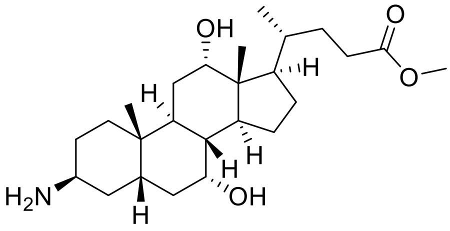

To a solution of methyl-3β-azido-7α,12α-dihydroxy-5β-cholan-24-oate (0.379 g, 0.847 mmol, 1 equiv.) in 10 mL tetrahydrofuran and 0.2 mL distilled water triphenylphosphine (0.334 g, 1.273 mmol, 1.5 equiv.) was added and the mixture was stirred for 18 h at 50 °C. The organic solvent was removed under reduced pressure and the crude product was purified by flash column chromatography (dichloromethane/methanol/triethylamine 10:1:0.1 to 5:1:0.1). The product was obtained as a white foam (0.327 g, 0.776 mmol, 92 %)
HRMS (ESI): m/z = 422.3267 $[M+Na]^+$ (calculated for 422.3265)
^{1}H-NMR ($CDCl_3$, 400.1 MHz): δ [ppm] = 4.03-3.92 (m, 1H), 3.88-3.78 (m, 1H), 3.65 (s, 3H), 3.30-3.21 (m, 1H), 2.59-2.46 (m, 1H), 2.44-2.31 (m, 1H), 2.29-2.09 (m, 3H), 1.99-1.62 (m, 10H), 1.58-1.25 (m, 12H), 1.21-1.05 (m, 1H), 0.97 (d, *J* = 6.1 Hz, 3H), 0.93 (s, 3H), 0.68 (s, 3H).
^{13}C-NMR ($CDCl_3$, 100.6 MHz): δ [ppm] = 174.88, 77.36, 73.11, 68.53, 53.13, 51.64, 47.34, 46.65, 42.04, 39.58, 35.97, 35.54, 35.36, 34.62, 31.27, 31.03, 29.97, 28.66, 27.62, 26.21, 23.39, 23.06, 17.48, 12.67, 8.28.

EXCLI Journal 2024;23:1330-1352 – ISSN 1611-2156
Received: August 01, 2024, accepted: October 28, 2024, published: October 30, 2024

Methyl-7α,12α-dihydroxy-3β-[(7-nitro-2,1,3-benzoxadiazol-4-yl)amino]-5β-cholan-24-oate S5

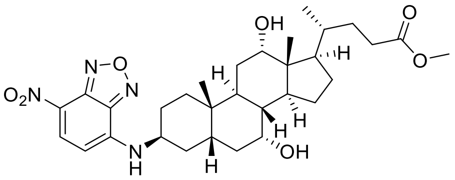

To a solution of methyl-3β-amino-7α,12α-dihydroxy-5β-cholan-24-oate (0.748 g, 1.774 mmol, 1 equiv.) in 50 mL methanol 4-chloro-7-nitrobenzo-2-oxa-1,3-diazol (0.569 g, 2.851 mmol, 1.6 equiv.) and sodium bicarbonate (0.328 g, 3.904 mmol, 2.2 equiv.) were added and the mixture was stirred for 18 h at 50 °C. The solvent was removed under reduced pressure and the crude product was purified by flash column chromatography (dichloromethane/acetone 10:1) to obtain the product as an orange solid (0.769 g, 1.315 mmol, 74 %).
HRMS (ESI): m/z = 607.3099 $[M+Na]^+$ (calculated for 607.3102)
^{1}H-NMR ($CDCl_3$, 400.1 MHz): δ [ppm] = 8.47 (d, *J* = 8.7 Hz, 1H), 6.40 (d, *J* = 7.1 Hz, 1H), 6.17 (d, *J* = 8.7 Hz, 1H), 4.12-3.99 (m, 1H), 3.96-3.86 (m, 1H), 3.67 (s, 3H), 2.87-2.72 (m, 1H), 2.43-2.33 (m, 1H), 2.30-2.19 (m, 2H), 2.09-2.00 (m, 1H), 1.99-1.12 (m, 22H), 1.02-0.96 (m, 6H), 0.72 (s, 3H).
^{13}C-NMR ($CDCl_3$, 100.6 MHz): δ [ppm] = 174.78, 144.62, 144.10, 143.11, 136.78, 123.75, 99.11, 72.97, 68.38, 51.69, 49.85, 47.50, 46.76, 42.08, 39.68, 37.39, 35.35, 35.25, 34.03, 32.63, 31.21, 31.06, 31.00, 28.72, 27.58, 26.40, 24.02, 23.30, 23.22, 17.53, 12.72.

Methyl-7α,12α-dihydroxy-3β-[(7-nitro-2,1,3-benzoxadiazol-4-yl)amino]-5β-cholan-24-oate

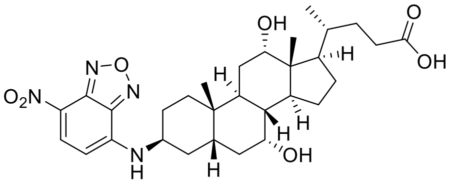

To a solution of methyl-7α,12α-dihydroxy-3β-[(7-nitro-2,1,3-benzoxadiazol-4-yl) amino]-5β-cholan-24-oate (0.127 g, 0.217 mmol, 1 equiv.) in 5 mL methanol a 2 N solution of lithium hydroxide (1.10 mL, 2.20 mmol, 10 equiv.) was added and the mixture was stirred for 3 h at 40 °C. The organic solvent was removed under reduced pressure and the residue was dissolved in 10 mL ethyl acetate and 5 mL 2 N HCl. The phases were separated, and the aqueous layer was extracted three times with 10 mL ethyl acetate. The combined organic layers were dried over $MgSO_4$ and the solvent was removed under reduced pressure. The crude product was purified by flash column chromatography (dichloromethane/methanol 9:1). The product was obtained as an orange solid (0.096 g, 0.168 mmol, 78 %)
HRMS (ESI): m/z = 593.2943 $[M+Na]^+$ (calculated for 593.2945)
^{1}H-NMR (DMSO-d_6, 400.1 MHz): δ [ppm] = 11.93 (s, 1H), 9.33-8.85 (m, 1H), 8.62-8.35 (m, 1H), 6.94-6.28 (m, 1H), 4.15 (t, *J* = 2.8 Hz, 1H), 4.13-3.99 (m, 1H), 3.79 (q, *J* = 3.1 Hz, 1H), 3.63 (t, *J* = 3.2 Hz, 1H), 3.17 (d, *J* = 4.9 Hz, 1H), 2.82-2.65 (m, 1H), 2.27-2.08 (m, 3H), 2.04-1.95 (m, 1H), 1.90-1.54 (m, 9H), 1.51-1.13 (m, 10H), 0.95-0.85 (m, 6H), 0.59 (s, 3H).
^{13}C-NMR (DMSO-d_6, 100.6 MHz): δ [ppm] = 174.99, 144.49, 144.23, 137.70, 120.72, 100.21, 88.06, 71.02, 66.26, 49.91, 46.11, 45.82, 41.38, 40.15, 39.41, 36.39, 35.05, 34.56, 34.15, 31.93, 30.83, 30.80, 28.68, 27.27, 26.16, 23.05, 22.78, 22.62, 16.96, 12.34.

EXCLI Journal 2024;23:1330-1352 – ISSN 1611-2156
Received: August 01, 2024, accepted: October 28, 2024, published: October 30, 2024

Tetrabutylammonium-2-[(tert-butoxycarbonyl)amino]ethane sulfonic acid S9

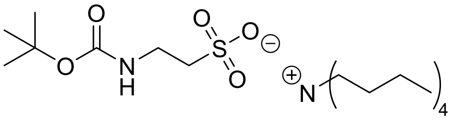

Taurine (0.252 g, 2.014 mmol, 1 equiv.) and 40 % aqueous tetrabutylammonium hydroxide (1.300 g, 2.004 mmol, 1 equiv.) were dissolved in 8 mL distilled water. Boc anhydride (0.439 g, 2.011 mmol, 1 equiv.) in 10 mL acetone was added dropwise. The mixture was stirred for 18 h at RT. Then the organic solvent was removed under reduced pressure. The aqueous residue was extracted three times with 20 mL dichloromethane and the combined organic layers were dried over $MgSO_4$. The solvent was removed under reduced pressure to obtain the product as a pale-yellow gel (0.897 g, 1.922 mmol, 96 %).
HRMS (ESI): m/z = 224.0603 $[M]^-$ (calculated for 224.0598)
HRMS (ESI): m/z = 242.2846 $[M]^+$ (calculated for 242.2842)
^{1}H-NMR ($CDCl_3$, 400.1 MHz): δ [ppm] = 3.53-3.48 (m, 2H), 3.28-3.17 (m, 8H), 2.91-2.84 (m, 2H), 1.64-1.52 (m, 8H), 1.44-1.36 (m, 8H), 1.34 (s, 9H), 0.95 (t, *J* = 7.3 Hz, 12H).
^{13}C-NMR ($CDCl_3$, 100.6 MHz): δ [ppm] = 156.16, 78.62, 58.95, 50.93, 37.15, 28.58, 24.15, 19.85, 13.79.

2,2,2-Trifluorethyl-2-[[1,1-dimethylethoxy) carbonyl] amino] ethane sulfonate S10

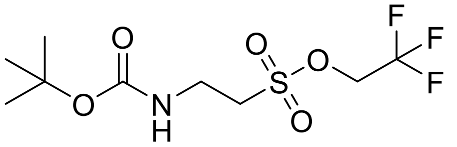

Under nitrogen atmosphere tetrabutylammonium-2-[(tert-butoxycarbonyl) amino] ethane-sulfonic acid (5.044 g, 10.807 mmol, 1 equiv.) was dissolved in 30 mL anhydrous dimethylformamide. Oxalyl chloride (1.10 mL, 12.86 mmol, 1.2 equiv.) in 10 mL anhydrous dichloromethane was added dropwise at 0 °C and the mixture was stirred for 1 h at 0 °C (mixture A). In a different flask triethylamine (2.23 mL, 16.07 mmol, 1.5 eq.) was dissolved in 20 mL dichloromethane under nitrogen atmosphere. Trifluoroethanol (0.93 mL, 12.86 mmol, 1.2 equiv.) was added dropwise at 0 °C and the mixture was stirred for 1 h at 0 °C (mixture B). Mixture A was added to mixture B dropwise at 0 °C and the resulting mixture was stirred for 18 h at RT. Then 30 mL distilled water were added, and the phases were separated. The aqueous layer was extracted three times with 20 mL diethyl ether. The combined organic layers were washed with 30 mL distilled water and then dried over $MgSO_4$. The solvent was removed under reduced pressure and the crude product was purified by flash column chromatography (ethyl acetate/cyclohexane 1:3) to obtain the product as a white solid (1.805 g, 5.874 mmol, 54 %).
HRMS (ESI): m/z = 330.0594 $[M+Na]^+$ = (calculated for 330.0593)
^{1}H-NMR ($CDCl_3$, 400.1 MHz): δ [ppm] = 5.07 (s, 1H), 4.52 (q, *J* = 7.9 Hz, 2H), 3.73-3.58 (m, 2H), 3.55-3.35 (m, 2H), 1.44 (s, 9H).
^{13}C-NMR ($CDCl_3$, 100.6 MHz): δ [ppm] = 155.69, 122.06 (q, *J* = 277.8 Hz), 80.48, 64.00 (q, *J* = 38.3 Hz), 51.50, 35.47, 28.40.

EXCLI Journal 2024;23:1330-1352 – ISSN 1611-2156
Received: August 01, 2024, accepted: October 28, 2024, published: October 30, 2024

2,2,2-Trifluorethyl-2-aminoethane sulfonate S11

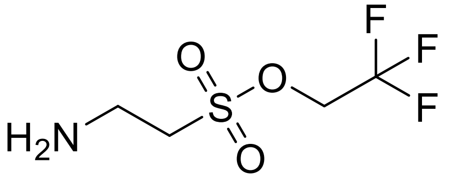

Trifluoro acetic acid (1.89 mL, 24.47 mmol, 10 equiv.) was added to a solution of 2,2,2-trifluorethyl-2-[[1,1-dimethylethoxy) carbonyl] amino] ethane sulfonate (0.752 g, 2.447 mmol, 1 equiv.) in 50 mL dichloromethane. The mixture was stirred for 4 h at RT. Then the solvent was removed under reduced pressure and the residue was dissolved in 20 mL ethyl acetate. The organic layer was washed three times with 20 mL 2 N sodium hydroxide and two times with brine. The organic layer was dried over $MgSO_4$, and the solvent was removed under reduced pressure to obtain the product as a colorless gel (0.465 g, 2.245 mmol, 92 %).
HRMS (ESI): m/z = 208.0250 $[M+H]^+$ (calculated for 208.0250)
^{1}H-NMR ($CDCl_3$, 400.1 MHz): δ [ppm] = 4.55 (q, *J* = 7.9 Hz, 2H), 3.40-3.33 (m, 2H), 3.31-3.16 (m, 2H), 1.43 (s, 2H).
^{13}C-NMR ($CDCl_3$, 100.6 MHz): δ [ppm] = 122.16 (q, *J* = 277.5 Hz), 63.92 (q, *J* = 38.2 Hz), 54.95, 36.92.

7α,12α-Dihydroxy-3β-[(7-nitro-2,1,3-benzoxadiazol-4-yl)amino]-5β-oxocholan-24-yl]amino] ethane trifluoroethane sulfonic acid ester S6

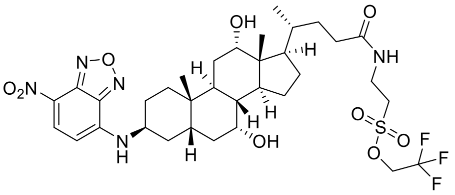

Under nitrogen atmosphere methyl-7α,12α-dihydroxy-3β-[(7-nitro-2,1,3-benzoxadiazol-4-yl)amino]-5β-cholan-24-oate (0.200 g, 0.350 mmol, 1 equiv.) was dissolved in 35 mL anhydrous dimethylformamide. Triethylamine (0.17 mL, 1.23 mmol, 3.5 equiv.), TBTU (0.169 g, 0.526 mmol, 1.5 equiv.), and HOBt (0.082 g, 0.535 mmol, 1.5 equiv.) were added and the mixture was stirred for 45 min at RT. Then 2,2,2-Trifluorethyl-2-aminoethane sulfonate (0.074 g, 0.357 mmol, 1 equiv.) in 5 mL dimethylformamide was added and the mixture was stirred for 18 h at RT. 20 mL distilled water were added to the mixture and the aqueous layer was extracted three times with 20 mL ethyl acetate. The combined organic layers were washed with 30 mL saturated sodium bicarbonate, potassium bisulfate, sodium bicarbonate and brine. The organic layer was dried over $MgSO_4$, and the solvent was removed under reduced pressure. The crude product was purified by flash column chromatography (ethyl acetate/cyclohexane 6:1) to obtain the product as an orange solid (0.245 g, 0.322 mmol, 92 %).
HRMS (ESI): m/z = 782.3015 $[M+Na]^+$ (calculated for 781.3017)
^{1}H-NMR ($CDCl_3$, 400.1 MHz): δ [ppm] = 8.48 (d, *J* = 8.6 Hz, 1H), 6.40 (d, *J* = 7.1 Hz, 1H), 6.24-6.19 (m, 1H), 6.18 (d, *J* = 8.7 Hz, 1H), 4.54 (q, *J* = 7.9 Hz, 2H), 4.11-4.03 (m, 1H), 4.03-4.00 (m, 1H), 3.95-3.87 (m, 1H), 3.77 (q, *J* = 5.8 Hz, 2H), 3.52-3.44 (m, 2H), 2.86-2.73 (m, 1H), 2.34-2.21 (m, 2H), 2.18-1.10 (m, 23H), 1.03-0.95 (m, 6H), 0.72 (s, 3H).
^{13}C-NMR ($CDCl_3$, 100.6 MHz): δ [ppm] = 174.23, 144.63, 144.12, 143.13, 136.81, 123.76, 122.06 (q, *J* = 277.7 Hz), 99.13, 77.37, 73.00, 68.37, 64.15 (q, *J* = 38.2 Hz), 51.05, 49.84, 47.21, 46.76, 42.12, 39.66, 37.40, 35.36, 35.33, 34.19, 34.02, 33.17, 32.62, 31.38, 31.07, 28.74, 27.63, 26.40, 24.03, 23.30, 23.23, 17.58.

EXCLI Journal 2024;23:1330-1352 – ISSN 1611-2156
Received: August 01, 2024, accepted: October 28, 2024, published: October 30, 2024

7α,12α-Dihydroxy-3β-[(7-nitro-2,1,3-benzoxadiazol-4-yl)amino]-5β-oxocholan-24yl]amino]ethane sulfonic acid S7

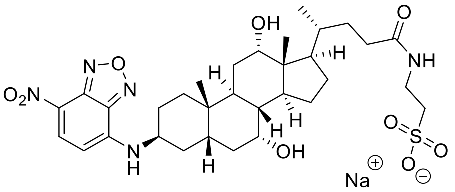

The 7α,12α-Dihydroxy-3β-[(7-nitro-2,1,3-benzoxadiazol-4-yl)amino]-5β-oxocholan-24-yl]amino] ethane trifluoroethane sulfonic acid ester (0.180 g, 0.237 mmol, 1 equiv.) was dissolved in 15 mL dichloromethane. Then 2 N sodium hydroxide (0.24 mL, 0.48 mmol, 2 equiv.) in methanol was added and the mixture was stirred for 3 h at RT. 10 mL distilled water were added, and the layers were separated. The organic layer was extracted three times with 10 mL distilled water. The combined aqueous layers were lyophilized, and the crude product was purified by flash column chromatography (dichloromethane/methanol 6:1). The product was obtained as an orange solid (0.058 g, 0.083 mmol, 35 %).
HRMS (ESI): m/z = 722.2806 $[M+Na]^+$ (calculated for 722.2806)
HRMS (ESI): m/z = 676.3026 $[M-Na]^-$ (calculated for 676.3022)
^{1}H-NMR (DMSO-d_6, 400.1 MHz): δ [ppm] = 9.37-8.71 (m, 1H), 8.54-8.39 (m, 1H), 7.69 (t, *J* = 5.5 Hz, 1H), 6.79-6.27 (m, 1H), 4.24-4.12 (m, 1H), 4.13-4.00 (m, 1H), 3.82-3.74 (m, 1H), 3.70-3.60 (m, 1H), 3.32-3.24 (m, 2H), 3.16 (d, *J* = 4.9 Hz, 1H), 2.73 (s, 1H), 2.60-2.52 (m, 2H), 2.30-2.14 (m, 1H), 2.14-1.91 (m, 3H), 1.87-1.49 (m, 10H), 1.49-1.34 (m, 5H), 1.30-1.12 (m, 4H), 0.97-0.86 (m, 6H), 0.59 (s, 3H).
^{13}C-NMR (DMSO-d_6, 100.6 MHz): δ [ppm] = 172.19, 144.60, 137.64, 127.71, 125.93, 107.12, 100.35, 71.02, 67.18, 66.26, 50.61, 48.59, 46.14, 45.81, 41.36, 39.43, 36.40, 35.46, 35.13, 34.57, 34.16, 32.72, 31.61, 29.08, 28.69, 27.27, 24.96, 22.78, 22.62, 19.98, 17.13, 12.36.

EXCLI Journal 2024;23:1330-1352 – ISSN 1611-2156
Received: August 01, 2024, accepted: October 28, 2024, published: October 30, 2024

^{1}H and ^{13}C NMR Spectra
Methyl-3α,7α,12α-trihydroxy-5β-cholan-24-oate

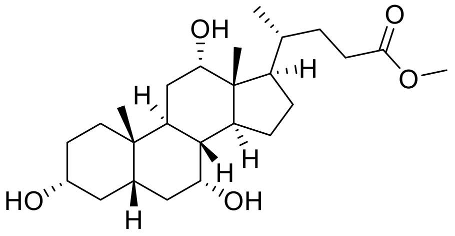

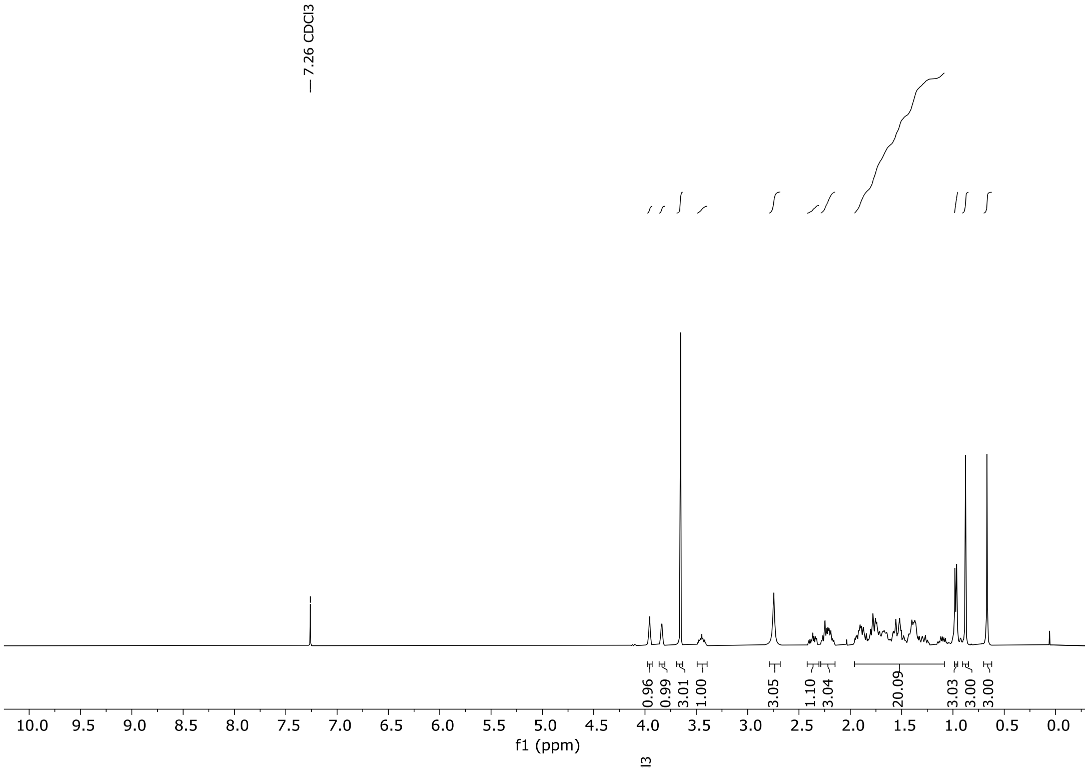

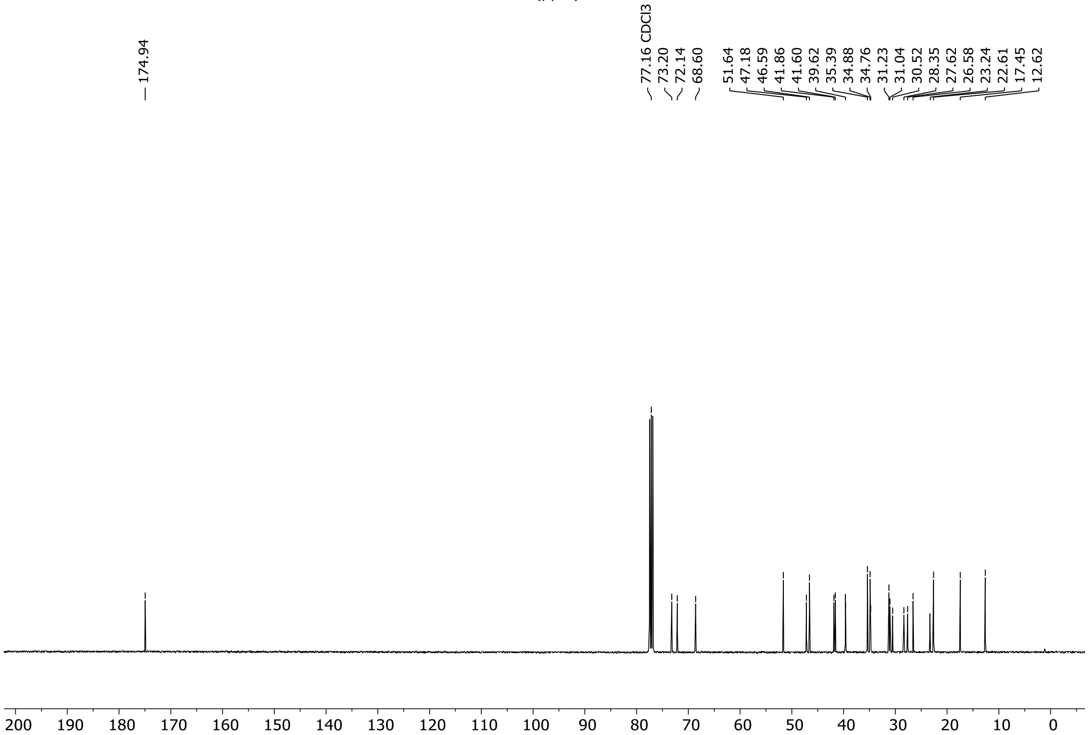

EXCLI Journal 2024;23:1330-1352 – ISSN 1611-2156
Received: August 01, 2024, accepted: October 28, 2024, published: October 30, 2024

Methyl-7α,12α-dihydroxy-3α-[(methylsulfonyl)oxy]-5β-cholan-24-oate S2

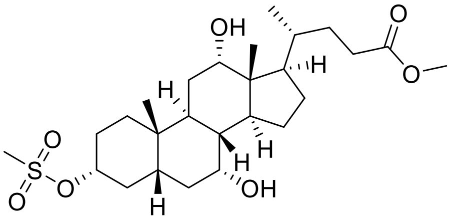

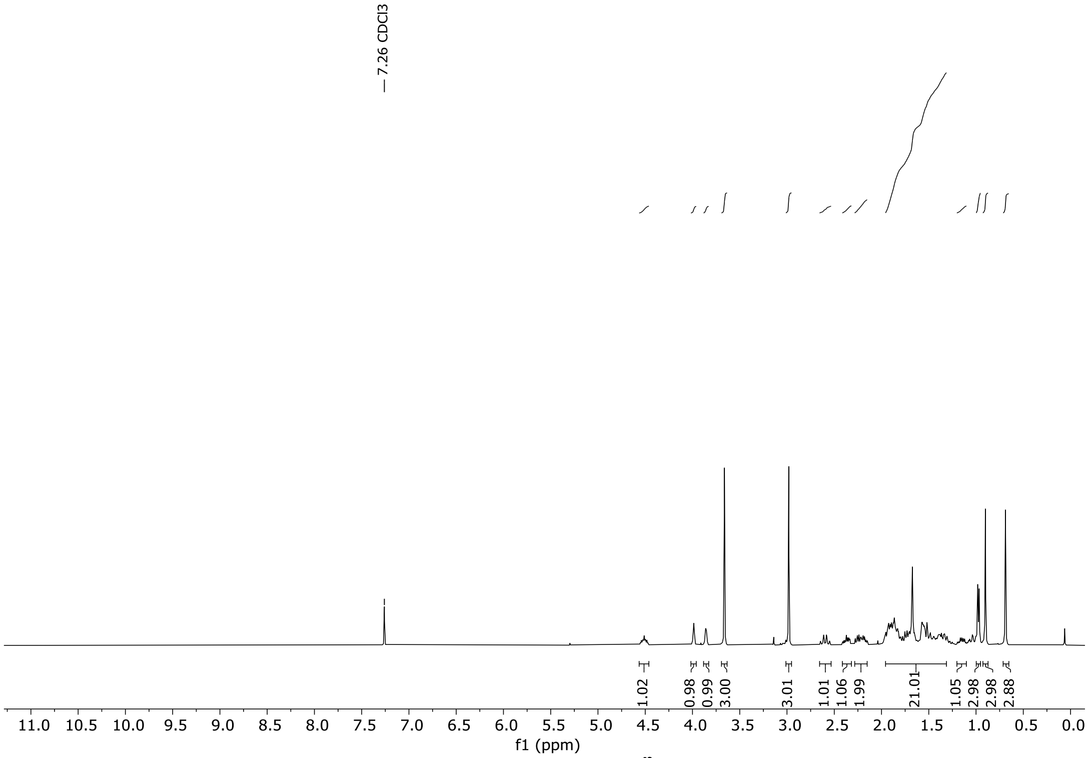

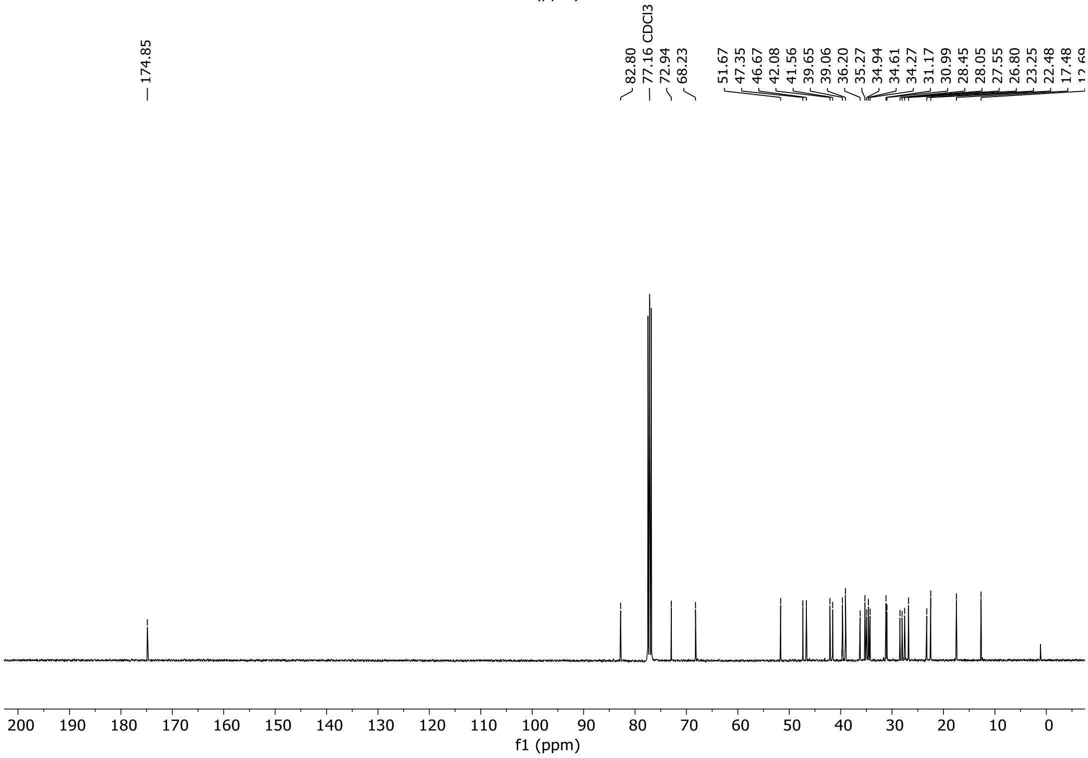

EXCLI Journal 2024;23:1330-1352 – ISSN 1611-2156
Received: August 01, 2024, accepted: October 28, 2024, published: October 30, 2024

Methyl-3β-azido-7α,12α-dihydroxy-5β-cholan-24-oate S3

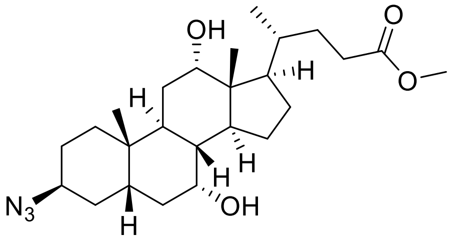

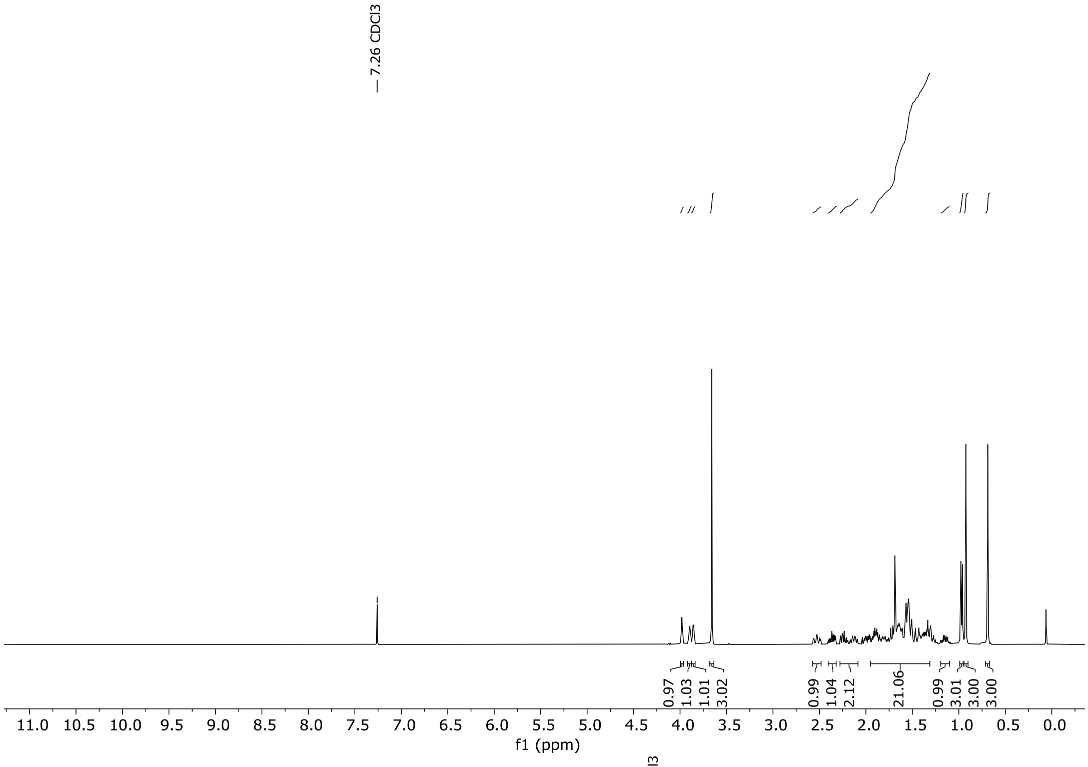

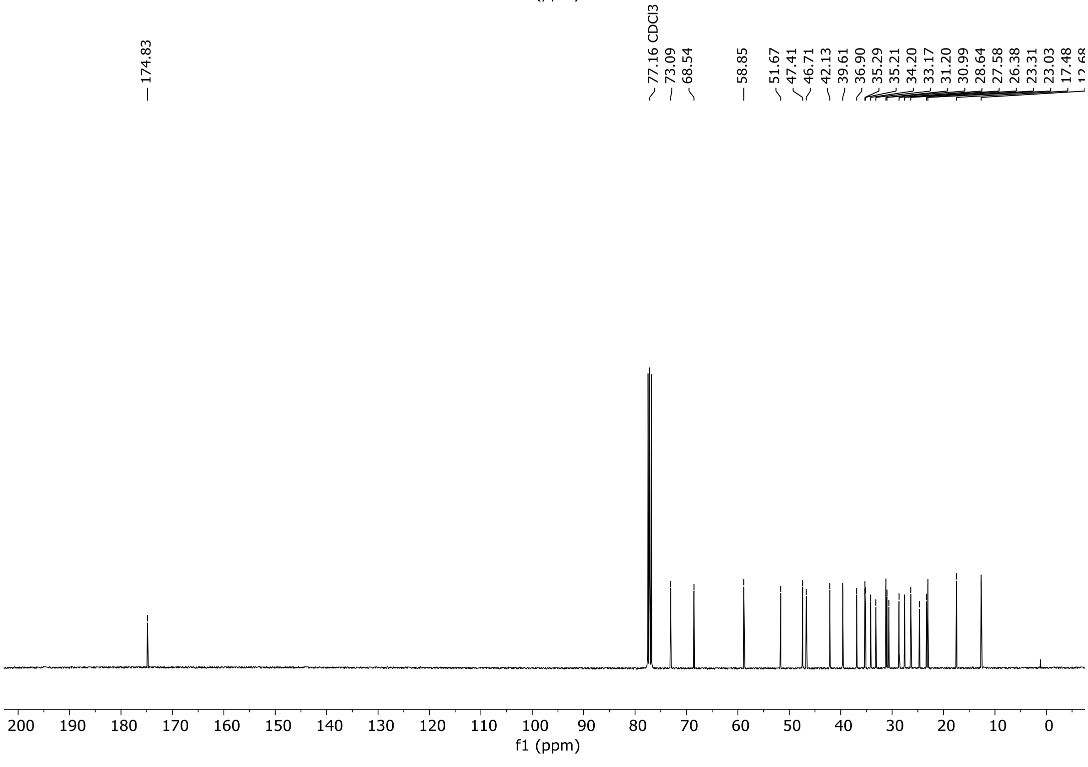

EXCLI Journal 2024;23:1330-1352 – ISSN 1611-2156
Received: August 01, 2024, accepted: October 28, 2024, published: October 30, 2024

Methyl-3β-amino-7α,12α-dihydroxy-5β-cholan-24-oate S4

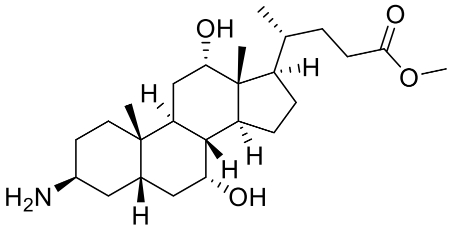

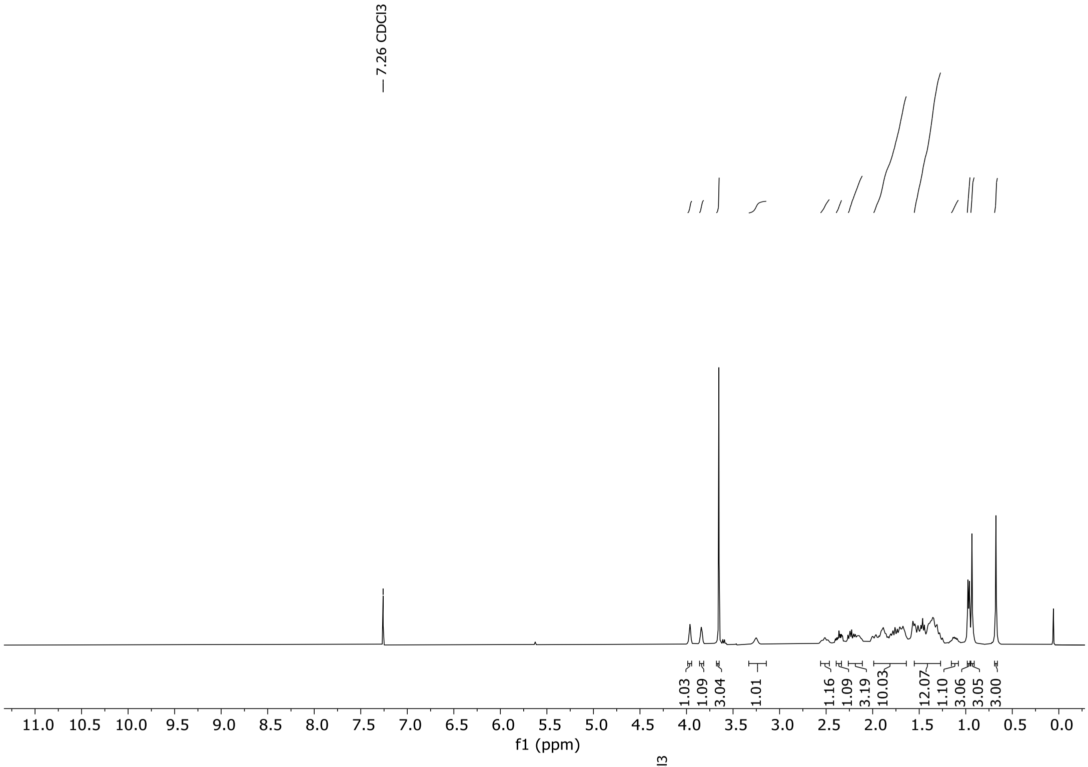

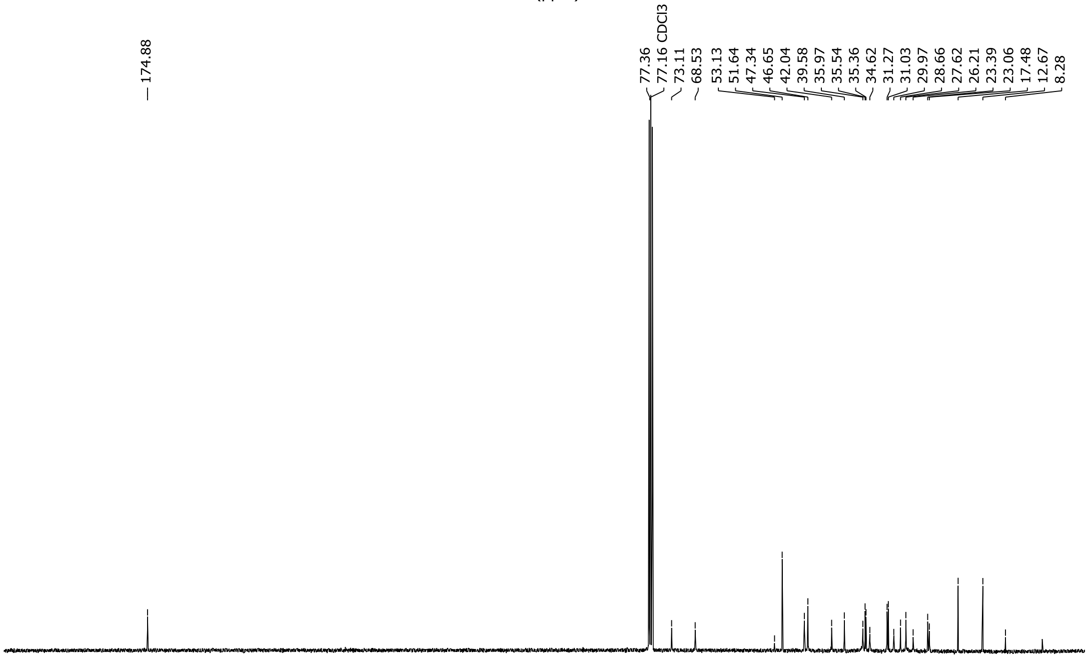

EXCLI Journal 2024;23:1330-1352 – ISSN 1611-2156
Received: August 01, 2024, accepted: October 28, 2024, published: October 30, 2024

Methyl-7α,12α-dihydroxy-3β-[(7-nitro-2,1,3-benzoxadiazol-4-yl)amino]-5β-cholan-24-oate S5

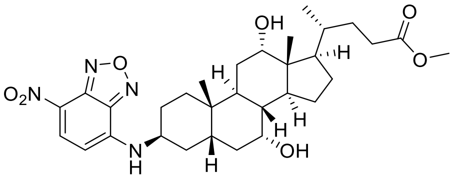

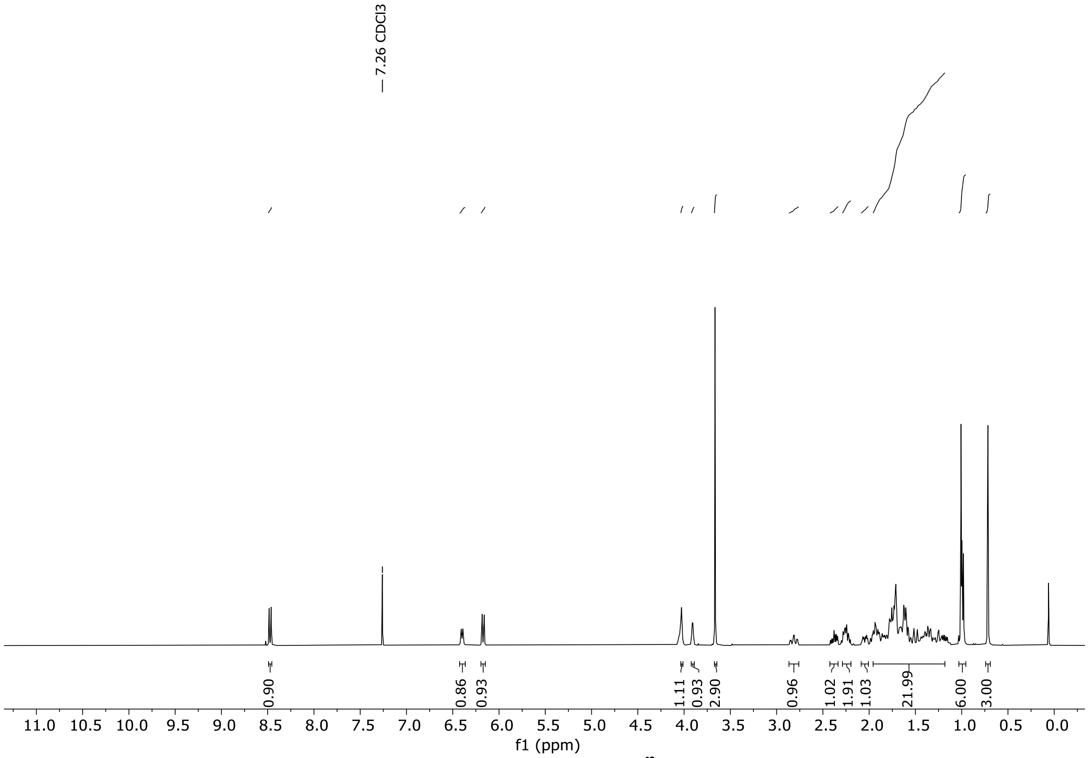

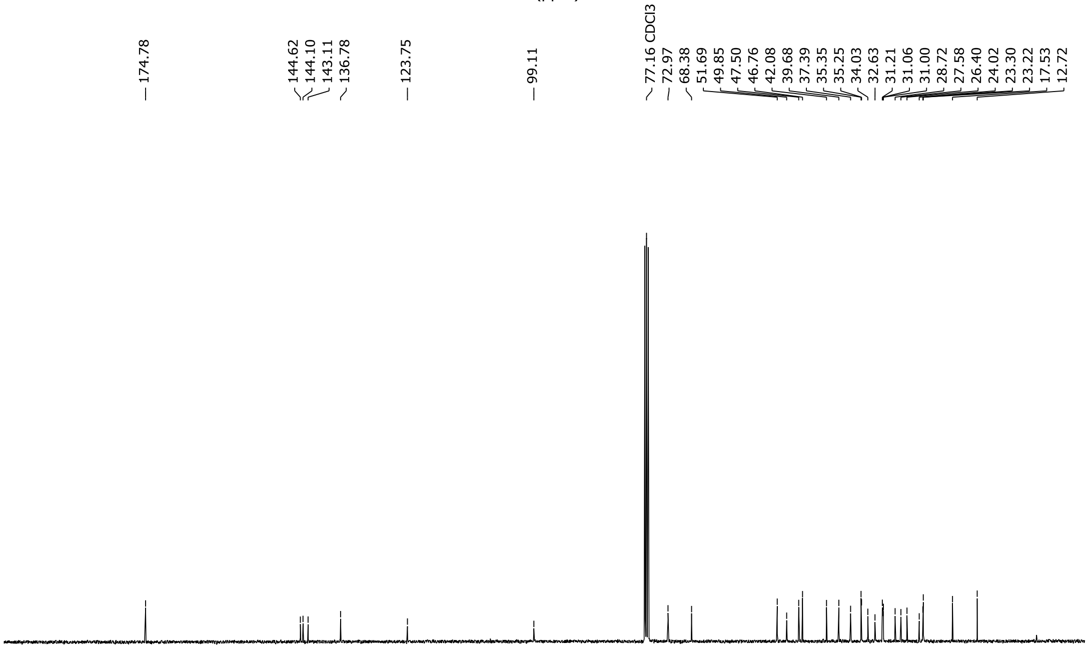

EXCLI Journal 2024;23:1330-1352 – ISSN 1611-2156
Received: August 01, 2024, accepted: October 28, 2024, published: October 30, 2024

Methyl-7α,12α-dihydroxy-3β-[(7-nitro-2,1,3-benzoxadiazol-4-yl)amino]-5β-cholan-24-oate

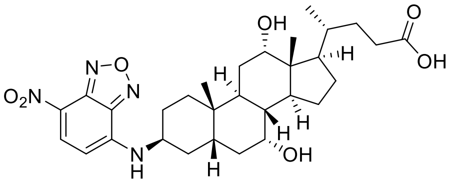

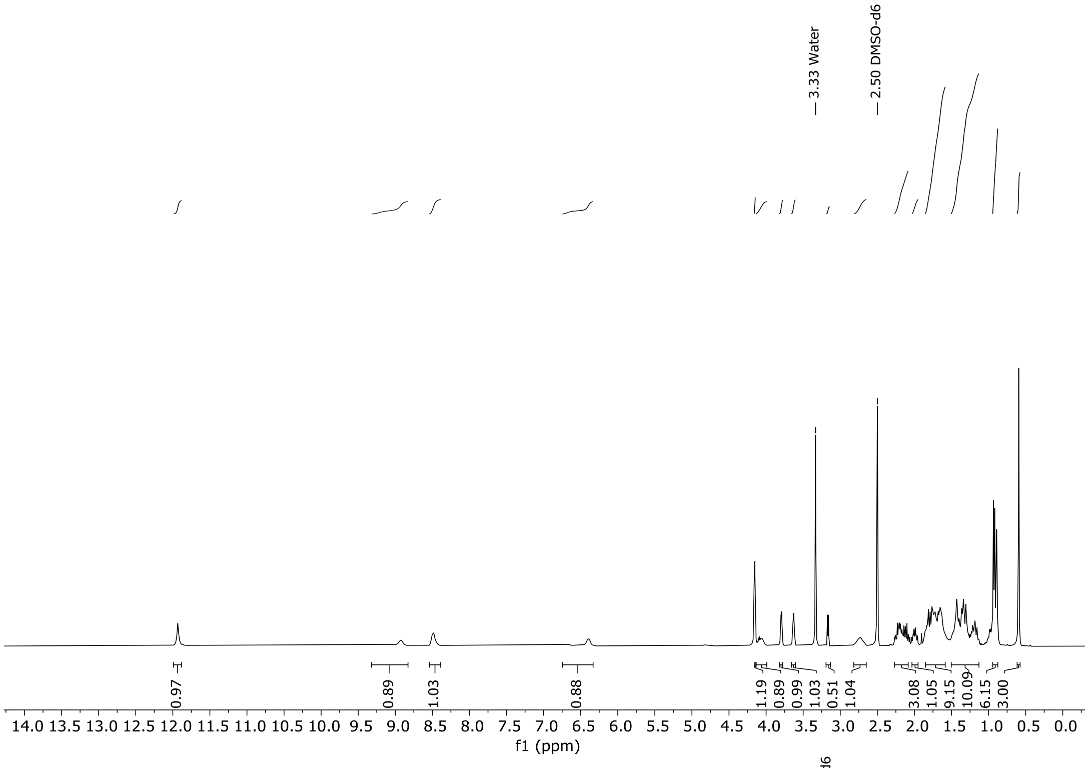

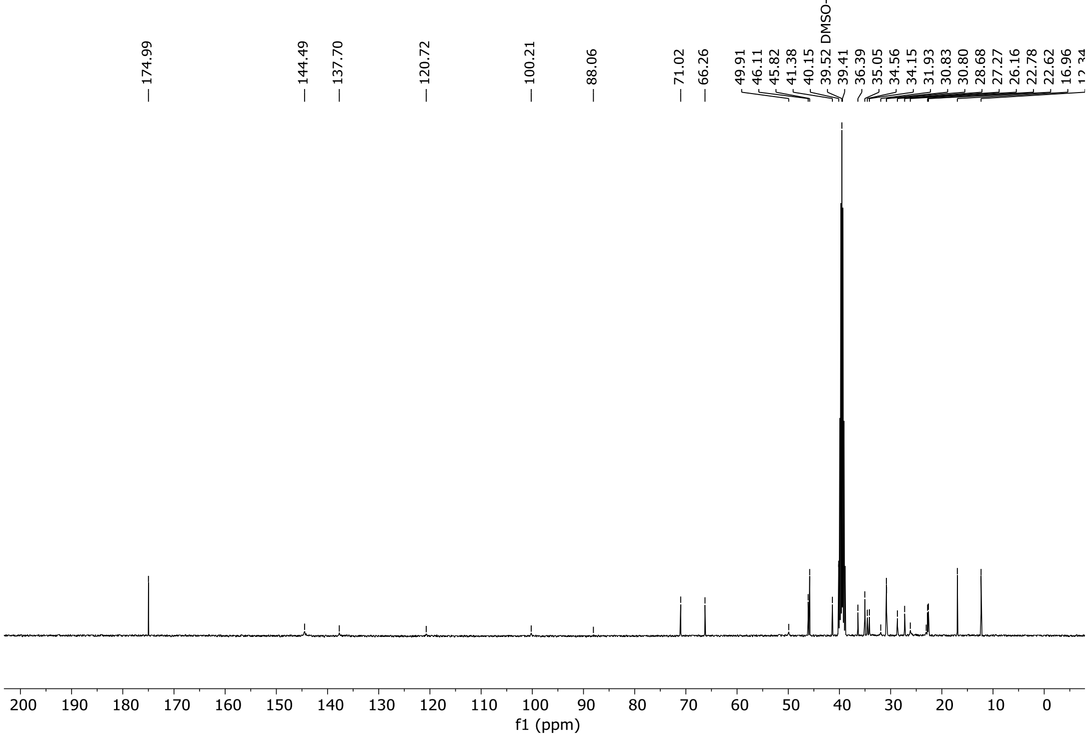

EXCLI Journal 2024;23:1330-1352 – ISSN 1611-2156
Received: August 01, 2024, accepted: October 28, 2024, published: October 30, 2024

Tetrabutylammonium-2-[(tert-butoxycarbonyl)amino]ethane sulfonic acid S9

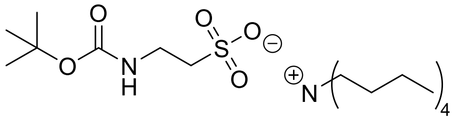

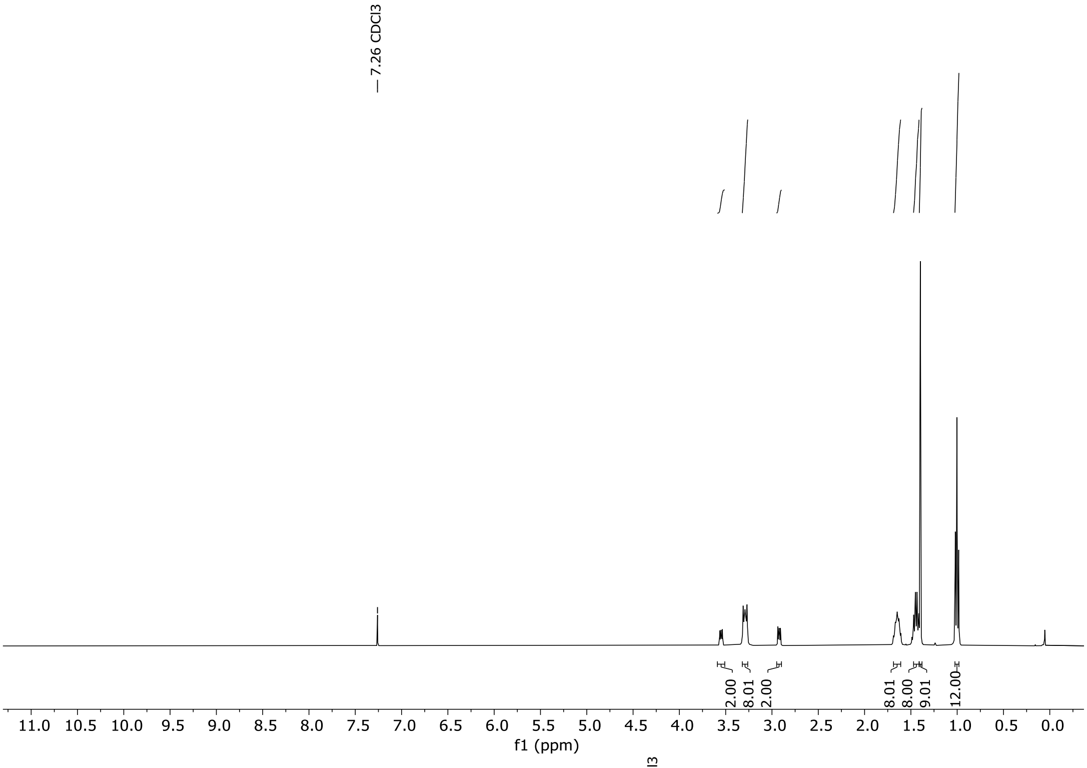

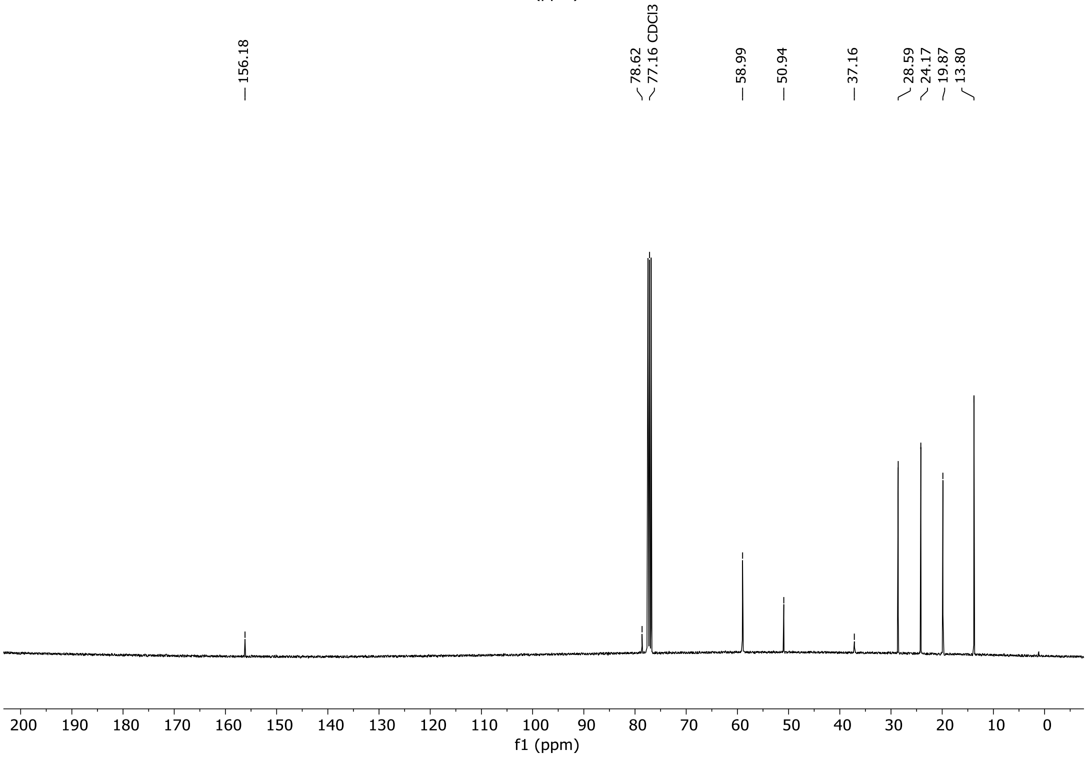

EXCLI Journal 2024;23:1330-1352 – ISSN 1611-2156
Received: August 01, 2024, accepted: October 28, 2024, published: October 30, 2024

2,2,2-Trifluorethyl-2-[[1,1-dimethylethoxy) carbonyl] amino] ethane sulfonate S10

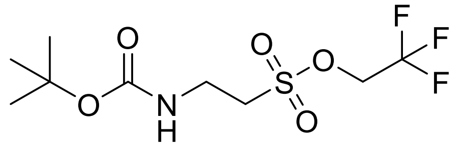

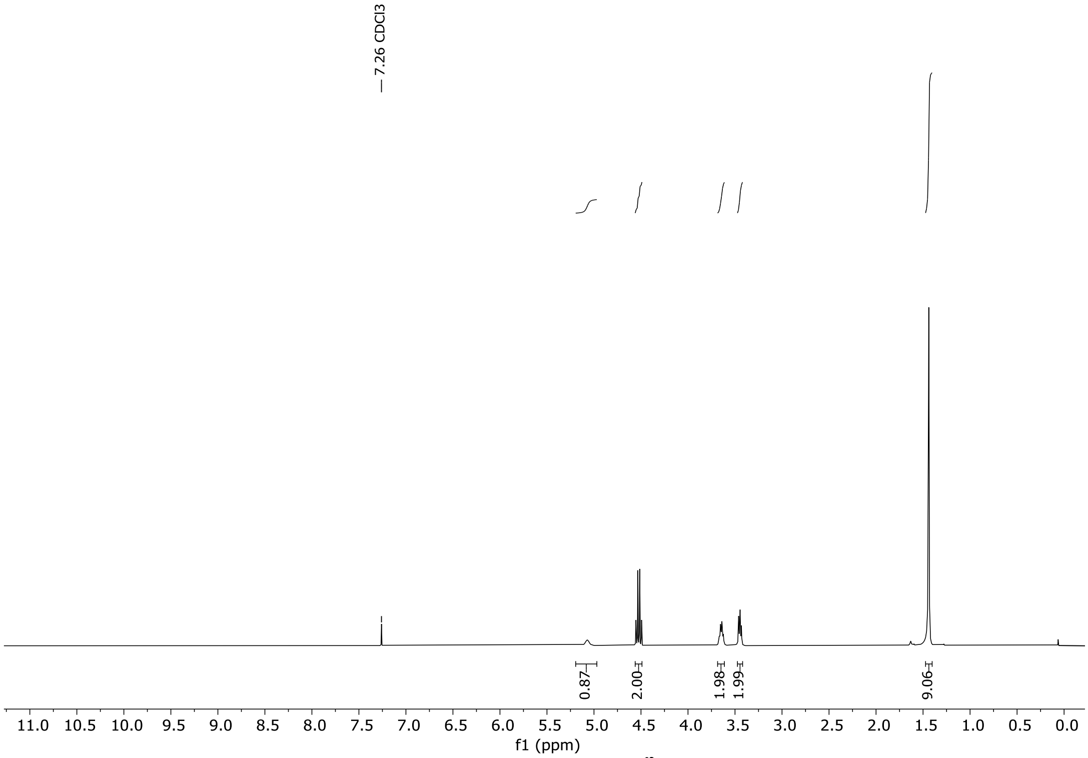

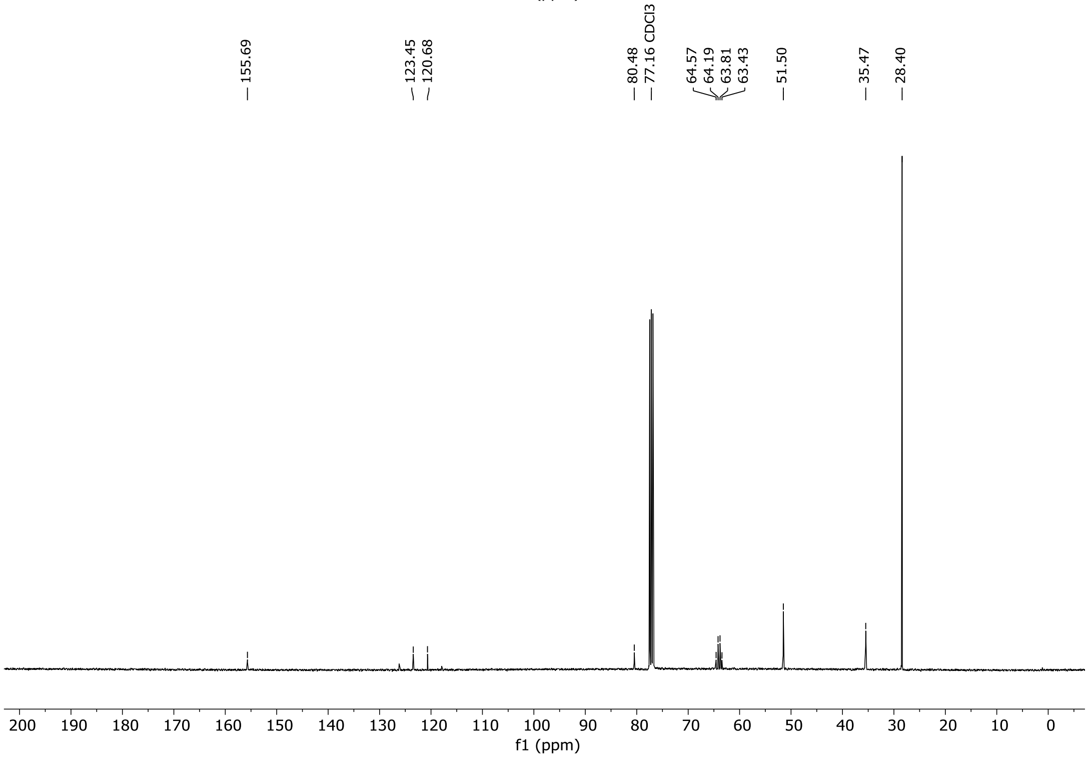

EXCLI Journal 2024;23:1330-1352 – ISSN 1611-2156
Received: August 01, 2024, accepted: October 28, 2024, published: October 30, 2024

2,2,2-Trifluorethyl-2-aminoethane sulfonate S11

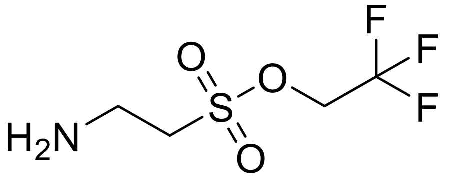

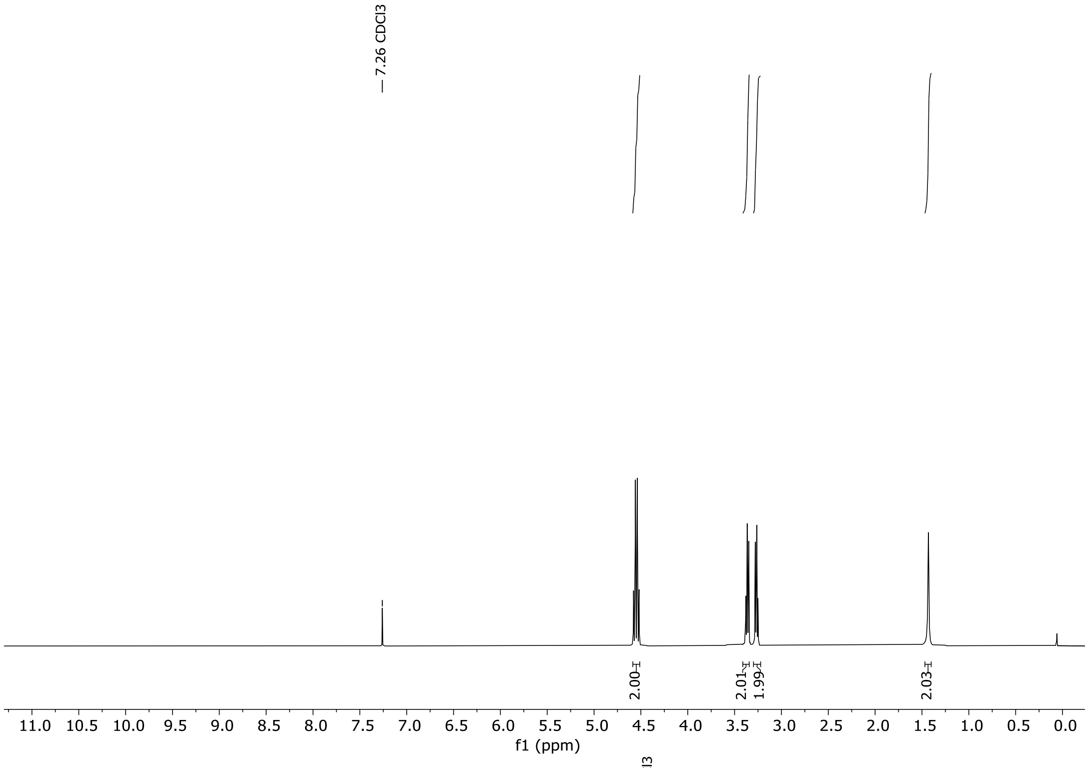

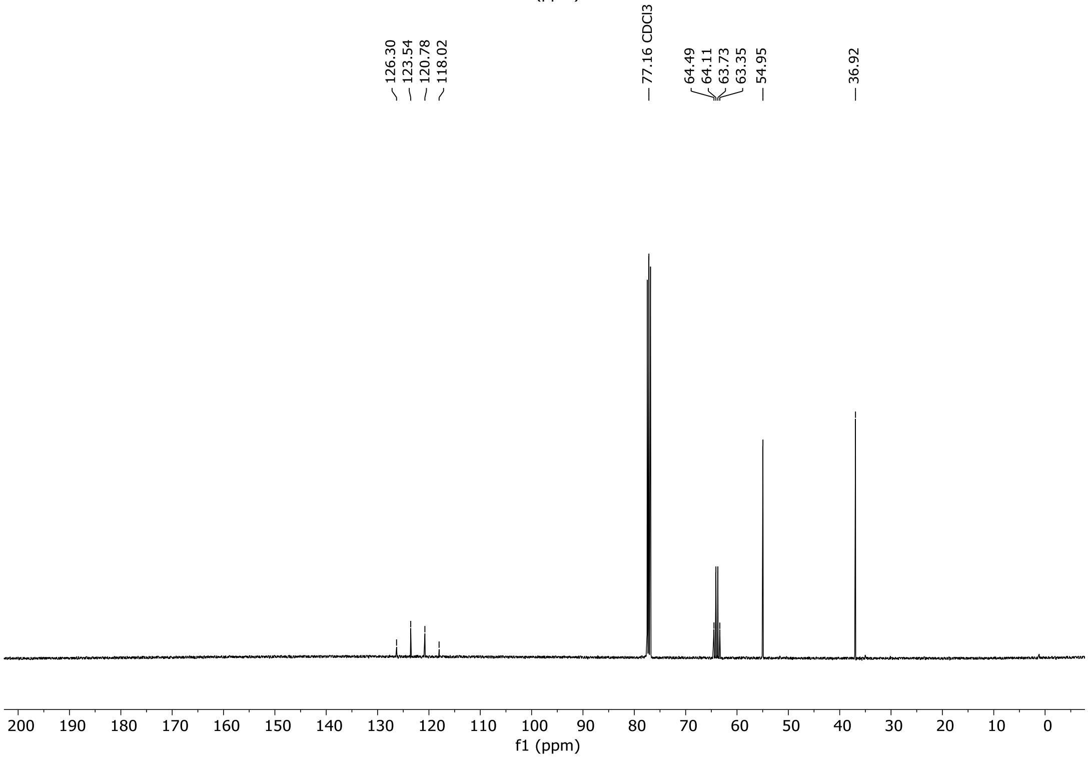

EXCLI Journal 2024;23:1330-1352 – ISSN 1611-2156
Received: August 01, 2024, accepted: October 28, 2024, published: October 30, 2024

7α,12α-Dihydroxy-3β-[(7-nitro-2,1,3-benzoxadiazol-4-yl)amino]-5β-oxocholane-24-yl]amino] ethane sulfonic acid S6

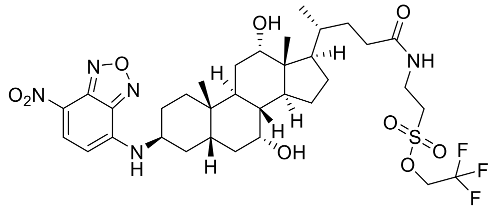

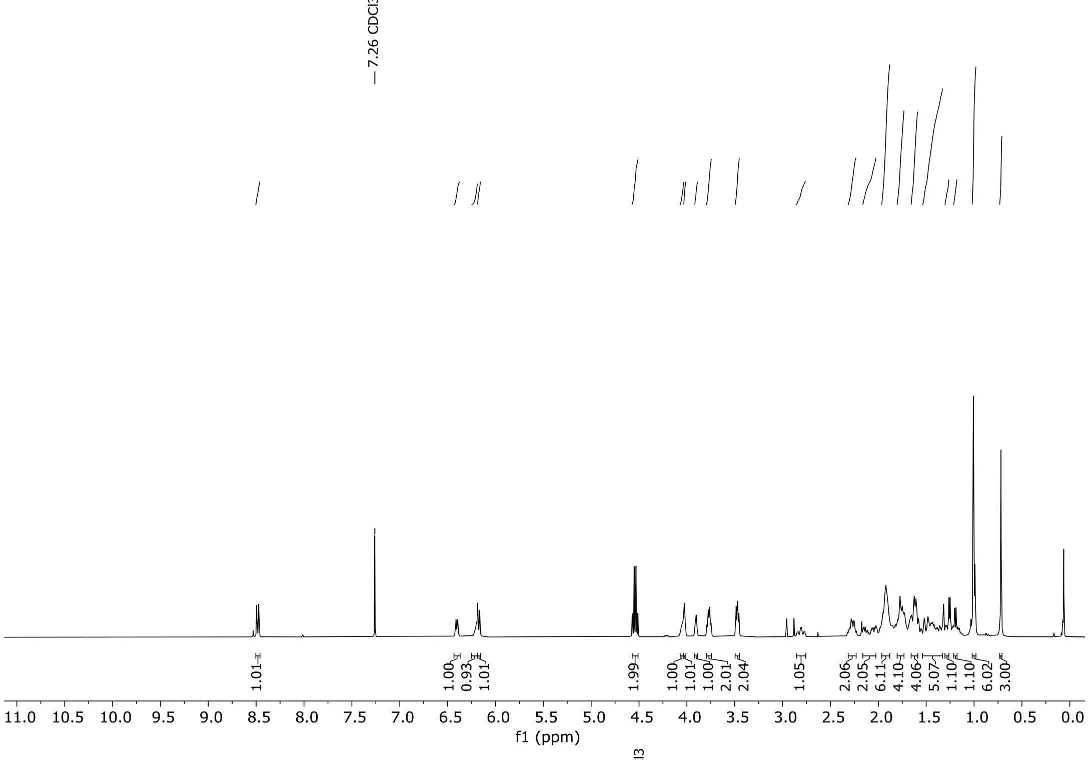

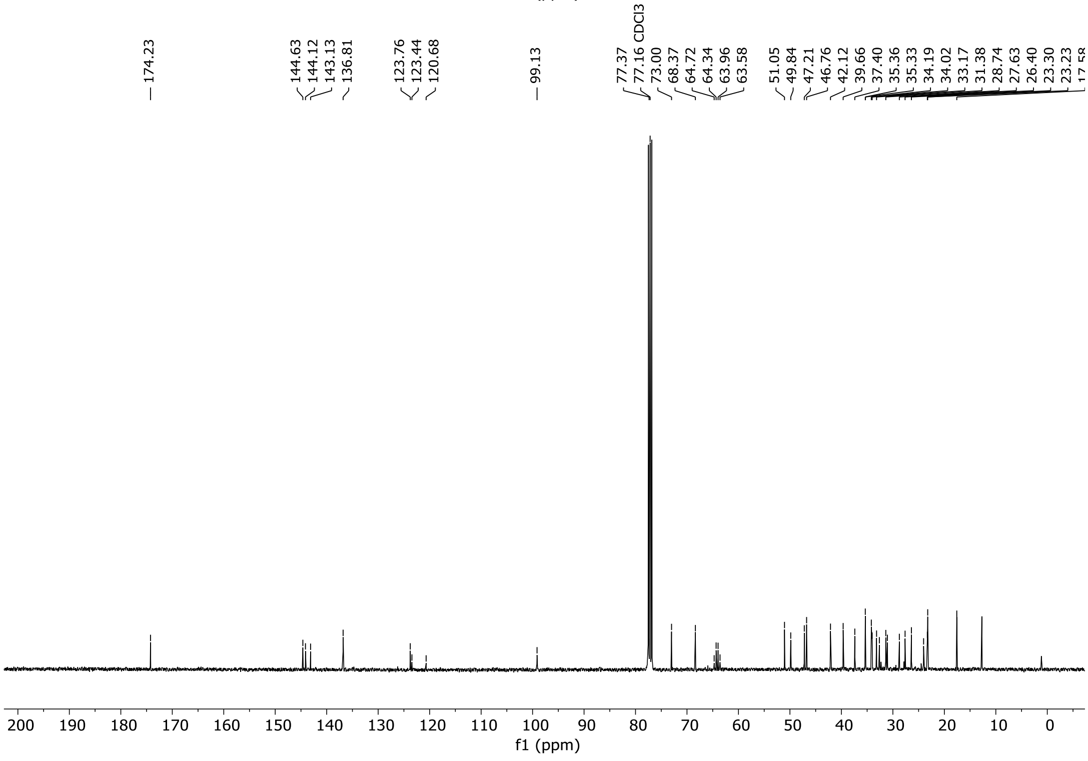

EXCLI Journal 2024;23:1330-1352 – ISSN 1611-2156
Received: August 01, 2024, accepted: October 28, 2024, published: October 30, 2024

7α,12α-Dihydroxy-3β-[(7-nitro-2,1,3-benzoxadiazol-4-yl)amino]-5β-oxocholane-24yl]amino]ethane sulfonic acid S7

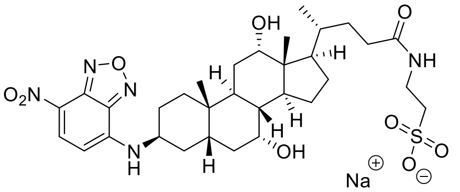

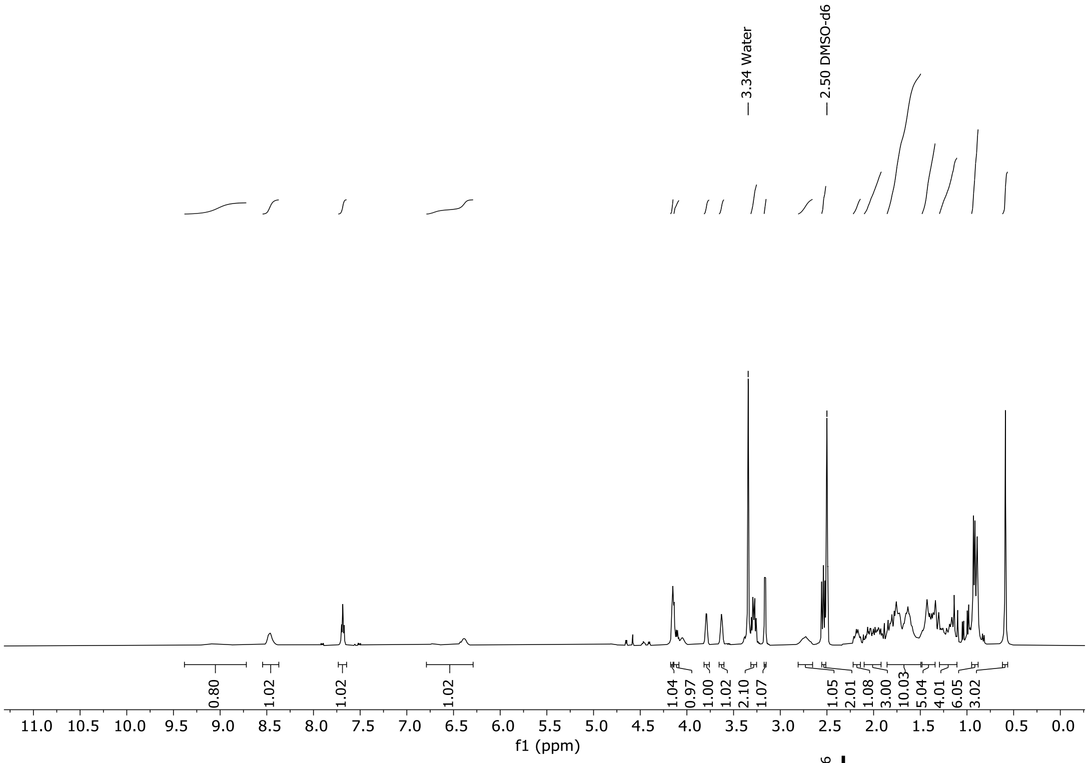

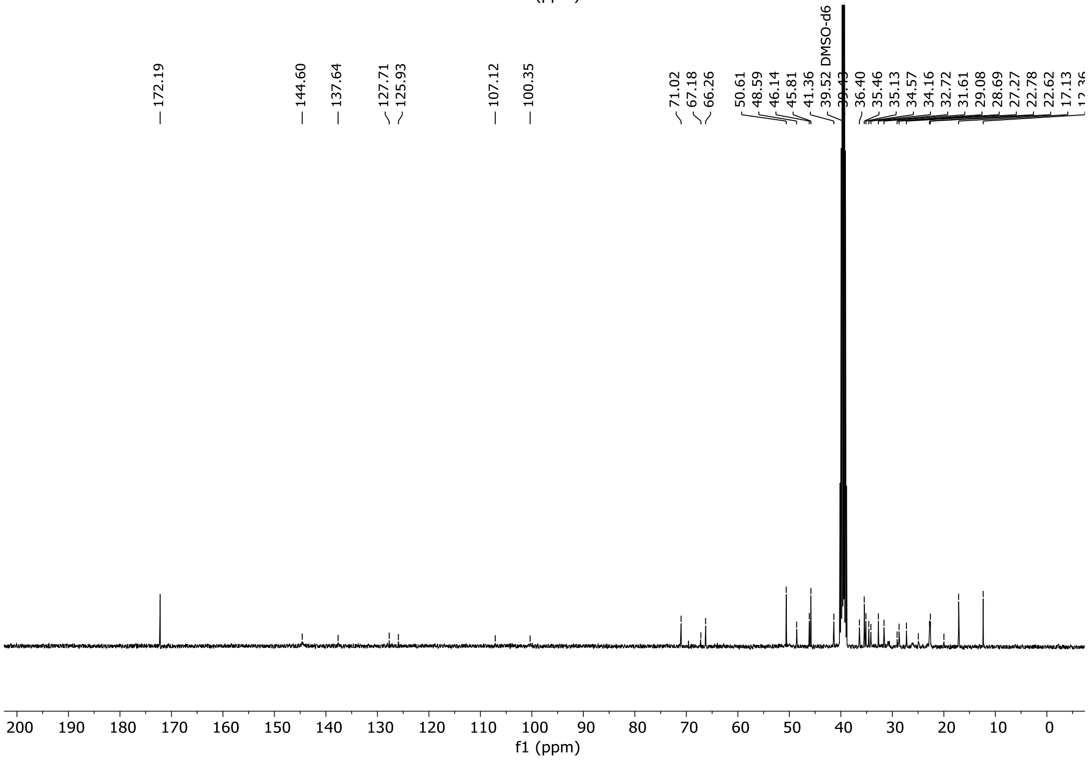

EXCLI Journal 2024;23:1330-1352 – ISSN 1611-2156
Received: August 01, 2024, accepted: October 28, 2024, published: October 30, 2024

SUPPLEMENTARY FIGURE

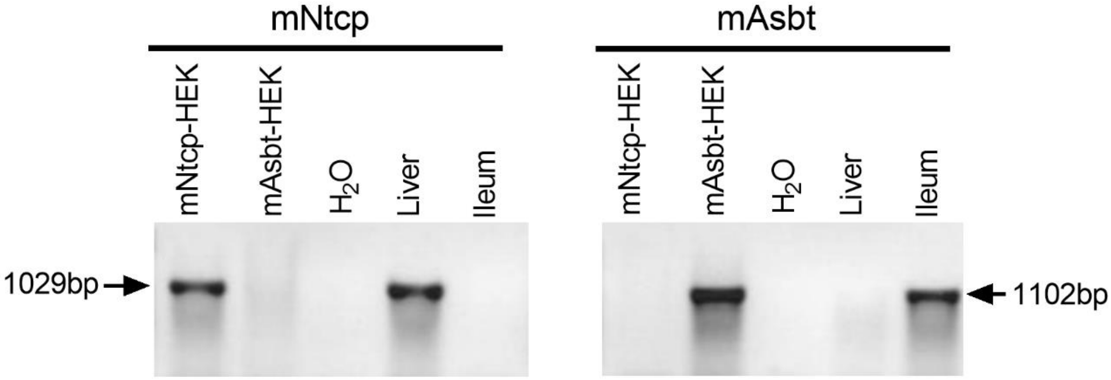

Supplementary Figure 1: PCR detection of mNtcp and mAsbt mRNA expression in mouse liver and ileum, respectively and in mNtcp-Hek and mAsbt-HEK cells
