# Supplementary material for: Validation of NBD-coupled taurocholic acid for intravital analysis of bile acid transport in liver and kidney of mice
Source: EXCLI J. 2024 Oct 30;23:1330–52. doi: 10.17179/excli2024-7707 (PMC11579514; doi:10.17179/excli2024-7707)
Supplement: Legends to supplementary videos [file EXCLI-23-1330-s-003.pdf]

EXCLI Journal 2024;23:1330-1352 – ISSN 1611-2156
Received: August 01, 2024, accepted: October 28, 2024, published: October 30, 2024

Legends to Supplementary videos

Original article:

VALIDATION OF NBD-COUPLED TAUROCHOLIC ACID FOR INTRAVITAL ANALYSIS OF BILE ACID TRANSPORT IN LIVER AND KIDNEY OF MICE

Ahmed Ghallab[1,2*], Sebastian Kunz[3], Celine Drossel[4], Veronica Billo[3], Adrian Friebel[5], Mats Georg[4], Richard Göttlich[4], Zaynab Hobloss[1], Reham Hassan[1,2], Maiju Myllys[1], Abdel-latief Seddek[2], Noha Abdelmageed[6], Paul A. Dawson[7], Erik Lindström[8], Stefan Hoehme[5], Jan G. Hengstler[1*#], Joachim Geyer[3*#]

[1] Department of Toxicology, Leibniz Research Centre for Working Environment and Human Factors, Technical University Dortmund, Ardeystr. 67, 44139 Dortmund, Germany
[2] Department of Forensic Medicine and Toxicology, Faculty of Veterinary Medicine, South Valley University, 83523 Qena, Egypt
[3] Institute of Pharmacology and Toxicology, Justus Liebig University Giessen, Biomedical Research Center Seltersberg, Schubertstr. 81, 35392 Giessen, Germany
[4] Institute of Organic Chemistry, Justus Liebig University Giessen, Heinrich-Buff-Ring 17, 35392, Giessen, Germany
[5] Institute of Computer Science & Saxonian Incubator for Clinical Research (SIKT), University of Leipzig, Haertelstraße 16-18, 04107 Leipzig, Germany
[6] Department of Pharmacology, Faculty of Veterinary Medicine, Sohag University, 82524 Sohag, Egypt
[7] Department of Pediatrics, Division of Gastroenterology, Hepatology, and Nutrition, Emory University, Atlanta, GA 30322, USA
[8] Albireo Pharma, Inc., Boston, MA 02109, USA

[#] Indicates shared senior authorship
* **Corresponding authors:** Ahmed Ghallab, Jan G. Hengstler, Department of Toxicology, Leibniz Research Centre for Working Environment and Human Factors, Technical University Dortmund, Ardeystr. 67, 44139 Dortmund, Germany. E-mail: ghallab@ifado.de; hengstler@ifado.de
Joachim Geyer, Institute of Pharmacology and Toxicology, Justus Liebig University Giessen, Biomedical Research Center Seltersberg, Schubertstr. 81, 35392 Giessen, Germany. E-mail: Joachim.M.Geyer@vetmed.uni-giessen.de

https://dx.doi.org/10.17179/excli2024-7707

This is an Open Access article distributed under the terms of the Creative Commons Attribution License (http://creativecommons.org/licenses/by/4.0/).

EXCLI Journal 2024;23:1330-1352 – ISSN 1611-2156
Received: August 01, 2024, accepted: October 28, 2024, published: October 30, 2024

Suppl. video 1: Kinetics of 3β-NBD-TCA in the liver of a WT mouse. Red: TMRE; green: 3β-NBD-TCA; blue: Hoechst 33258. The data corresponds to **Figure 5** of the main manuscript.

Suppl. video 2: Kinetics of 3β-NBD-TCA in the liver of a WT mouse treated with Myrcludex B. Red: TMRE; green: 3β-NBD-TCA; blue: Hoechst 33258. The data corresponds to **Figure 5** of the main manuscript.

Suppl. video 3: Kinetics of 3β-NBD-TCA in the liver of an mOatp1a/1b cluster knockout mouse. Red: TMRE; green: 3β-NBD-TCA; blue: Hoechst 33258. The data corresponds to **Figure 6** of the main manuscript.

Suppl. video 4: Kinetics of 3β-NBD-TCA in the liver of an mOatp1a/1b cluster knockout mouse treated with Myrcludex B. Red: TMRE; green: 3β-NBD-TCA; blue: Hoechst 33258. The data corresponds to **Figure 6** of the main manuscript.

Suppl. video 5: Kinetics of 3β-NBD-TCA in kidneys of an mOatp1a/1b cluster knockout mouse. Red: TMRE; green: 3β-NBD-TCA; blue: Hoechst 33258. The data corresponds to **Figure 7** of the main manuscript.

Suppl. video 6: Kinetics of 3β-NBD-TCA in kidneys of an mOatp1a/1b cluster knockout mouse treated with Myrcludex B. Red: TMRE; green: 3β-NBD-TCA; blue: Hoechst 33258. The data corresponds to **Figure 7** of the main manuscript.

Suppl. video 7: Kinetics of 3β-NBD-TCA in kidneys of an mOatp1a/1b cluster knockout mouse treated with Myrcludex B and AS0369. Red: TMRE; green: 3β-NBD-TCA; blue: Hoechst 33258. The data corresponds to **Figure 9** of the main manuscript.
